# Ambulatory oxygen in fibrotic lung disease (AmbOx): study protocol for a randomised controlled trial

**DOI:** 10.1186/s13063-017-1912-9

**Published:** 2017-04-28

**Authors:** Dina Visca, Vicky Tsipouri, Letizia Mori, Ashi Firouzi, Sharon Fleming, Morag Farquhar, Elizabeth Leung, Toby M. Maher, Paul Cullinan, Nick Hopkinson, Athol U. Wells, Winston Banya, Jennifer A. Whitty, Huzaifa Adamali, Lisa G. Spencer, Piersante Sestini, Elisabetta A. Renzoni

**Affiliations:** 1 Interstitial Lung Disease Unit, Royal Brompton Hospital, Imperial College, London, UK; 2 NIHR Respiratory Biomedical Research Unit, Royal Brompton Hospital, Sydney Street, London, UK; 3 Department of Public Health and Primary Care, University of Cambridge, Cambridge, UK; 4 Department of Occupational and Environmental Medicine, Royal Brompton Hospital and Imperial College (NHLI), London, UK; 5 Health Economics Group, Norwich Medical School, University of East Anglia, Norwich, UK; 6 Bristol Interstitial Lung Disease (BILD) Service, Southmead Hospital, Bristol, UK; 7 Aintree Chest Centre, University Hospital Aintree, Lower Lane, Liverpool, UK; 8 Department of Respiratory Medicine, Surgery and Neurosciences, University of Siena, Siena, Italy

**Keywords:** Fibrotic interstitial lung disease, Ambulatory oxygen, Oxygen desaturation, Health status, Quality of life, Activity measures, Shortness of breath

## Abstract

**Background:**

Fibrotic interstitial lung diseases (ILDs) are chronic and often progressive conditions resulting in substantial morbidity and mortality. Shortness of breath, a symptom often linked to oxygen desaturation on exertion, is tightly linked to worsening quality of life in these patients. Although ambulatory oxygen is used empirically in their treatment, there are no ILD-specific guidelines on its use. To our knowledge, no studies are available on the effects of ambulatory oxygen on day-to-day life in patients with ILD.

**Methods/design:**

Ambulatory oxygen in fibrotic lung disease (AmbOx) is a multicentre, randomised controlled crossover trial (RCT) funded by the Research for Patient Benefit Programme of the National Institute for Health Research. The trial will compare ambulatory oxygen used during daily activities with no ambulatory oxygen in patients with fibrotic lung disease whose oxygen saturation (SaO_2_) is ≥94% at rest, but drops to ≤88% on a 6-min Walk Test. The randomised controlled trial (RCT) will evaluate the effects on health status (measured by the King’s Brief ILD Questionnaire: K-BILD) of ambulatory oxygen used at home, at an optimal flow rate determined by titration at screening visit, and administered for a 2-week period, compared to 2 weeks off oxygen. Key secondary outcomes will include breathlessness on activity scores, as measured by the University of California San Diego Shortness of Breath Questionnaire, global patient assessment of change scores, as well as quality of life scores (St George’s Respiratory Questionnaire), anxiety and depression scores (Hospital Anxiety and Depression Scale), activity markers measured by SenseWear Armbands, pulse oximetry measurements, patient-reported daily activities, patient- and oxygen company-reported oxygen cylinder use. The study also includes a qualitative component and will explore in interviews patients’ experiences of the use of a portable oxygen supply and trial participation in a subgroup of 20 patients and carers.

**Discussion:**

This is the first RCT of the effects of ambulatory oxygen during daily life on health status and breathlessness in fibrotic lung disease. The results generated should provide the basis for setting up ILD-specific guidelines for the use of ambulatory oxygen.

**Trial registration:**

National Clinical Trials Registry, identifier: NCT02286063. Registered on 8 October 2014 (retrospectively registered).

**Electronic supplementary material:**

The online version of this article (doi:10.1186/s13063-017-1912-9) contains supplementary material, which is available to authorized users.

## Background

The hallmark symptom of fibrotic interstitial lung diseases (ILD) is breathlessness, which progressively limits the ability of patients to carry out routine activities and ultimately can affect their independence. Breathlessness is the main determinant of quality of life in patients with idiopathic pulmonary fibrosis (IPF), the most common of the idiopathic ILDs [1]. Fibrotic lung diseases profoundly affect an individual’s sense of wellbeing, with anxiety, depression and fatigue often accompanying exertional limitation, leading to a loss of health status. With limited pharmacological therapy proven to prolong survival in many ILDs, attempts to improve or maintain health status are of crucial importance.

Breathlessness on exertion, formally measured by the 6-min Walk Test (6MWT) [2], is often associated with a fall in oxygen saturation (SaO_2_), which in ILD patients can be particularly marked [3–6]. The 6MWT is a widely used field test measuring functional exercise capacity, repeatedly observed to have good validity as a functional and prognostic marker in IPF [7, 8]. Ambulatory oxygen used during a 6MWT or bicycle endurance test has been reported to improve breathlessness, distance walked, and breathlessness recovery time [9, 10]. Frank and coauthors reported that further up-titration of ambulatory oxygen in order to maintain saturation values above 90% and/or reaching a 6-L/min flow rate during a 6MWT seemed to improve exercise capacity in IPF, as measured by a 6MWT, even in patients already using oxygen at home [11]. However, two studies have failed to report a significant benefit on distance walked or dyspnoea of supplemental oxygen on either the 6MWT [12] or a shuttle endurance test [13], possibly because neither adequately titrated oxygen requirements to appropriately correct the patient’s exertional desaturation, as suggested in a recent Cochrane review [14].

Although a number of chest physicians prescribe ambulatory oxygen to ILD patients with desaturation on exercise, there are no nationally recognised guidelines to direct ambulatory oxygen prescription. In particular, NICE guidelines for oxygen use only address chronic obstructive pulmonary disease (COPD): (http://guidance.nice.org.uk/CG101), a disease which markedly differs from IPF or other ILDs. The recent British Thoracic Society (BTS) oxygen guidelines only briefly touch on ambulatory oxygen and give no specific guidance on its use in ILD, indeed recommending against its use in most cases [15]. Specifically, there are no published studies investigating the effects of ambulatory oxygen on day-to-day life in patients with fibrotic ILD, or assessing whether oxygen-induced improvements in 6MWT performance predict response to supplemental oxygen during activities of daily living, and an overall improvement in health status.

We have, therefore, planned a prospective study to assess whether individuals with fibrotic ILD whose SaO_2_ falls to ≤88% on a 6MWT, but are not hypoxic at rest, benefit from the use of ambulatory oxygen in their daily lives, by assessing changes in health status. The proposed project is the first of its kind in ILD, and addresses an under-researched area in urgent need of study. The study has the potential to lead to significant advances in the treatment and the understanding of exercise limitation in patients with ILD, and will represent an essential step towards the development of guidelines on oxygen use in ILD.

### Objectives

The main aim of this project is to establish whether ambulatory oxygen in patients with fibrotic ILD whose oxygen saturation falls to ≤88% on a 6MWT, leads to a significant improvement in health status, as measured by the King’s Brief Interstitial Lung Disease Questionnaire (K-BILD) [16]. Key additional outcomes will be the evaluation of dyspnoea during activities of daily living as measured by the University of California San Diego Shortness of Breath Questionnaire (UCSD SOBQ) [17], as well as the global patient assessment of change [18]. Further secondary outcomes will include monitored (SenseWear Armbands) and patient-recorded activity parameters, as well as quality of life scores assessed by the St. George’s Respiratory Questionnaire (SGRQ) [19] and the Hospital Anxiety and Depression Scale (HADS) [20]. We also aim to assess whether the improvement in 6MWT performance induced by portable oxygen can predict benefit of ambulatory oxygen in day-to-day living, by a placebo air-controlled 6MWT performed at the start of the study, to assess changes to the 6MWT parameters induced by supplemental oxygen. An economic evaluation will assess the potential value for money provided by ambulatory oxygen by comparing the health system costs and health-related benefits to those of no ambulatory oxygen.

In addition, qualitative semistructured interviews at the end of the 4-week period in a subgroup of 20 patients will be conducted to evaluate patients’ and carers’ experiences regarding the use of ambulatory oxygen and trial participation.

## Methods/design

The AmbOx study is a UK, multicentre, prospective, randomised controlled crossover trial of ambulatory oxygen against no ambulatory oxygen over a 4-week period (2 weeks on ambulatory oxygen and 2 weeks on no portable oxygen), to evaluate the effects of ambulatory oxygen on health status in patients with ILD. Patients whose SaO_2_ at rest is ≥94% will be screened by performing a 6MWT as part of their routine clinical assessment in order to identify those patients whose SaO_2_ drops to ≤88%. In those who desaturate below this threshold, a further 6MWT will be conducted with titration of oxygen flow so that for least half of the 6MWT, oxygen saturation with supplemental oxygen is maintained at >90% (for more detailed information on oxygen titration, see section 10.2 of the protocol (Additional file 1).

Following consent to the study, patients meeting eligibility criteria and deemed clinically stable at the end of the 2-week run-in period, and not expecting to require any change in their background medication, will be assigned in random order to 2 weeks on ambulatory oxygen or no oxygen during the baseline visit. Optimal oxygen flow rates will be determined during the screening visit on the basis of the entry 6MWT on oxygen, as detailed above. At the start of the active part of the trial, the effects of ambulatory oxygen on 6MWT performance will be formally evaluated by performing two 6MWTs, one on oxygen and one on placebo air-filled canisters, at the flow rate identified during the screening visit, in random order, with a rest of at least 30 min between tests. The patient will be blind to the content of the canisters. Measured parameters will include 6MWT distance, oxygen saturation and heart rate measured continuously (WristOx2™ model 3150), Borg Dyspnoea and Fatigue Score [21] before and at the end of the test, time to recovery of heart rate, and oxygen saturation. These parameters will be related to changes in the primary and secondary outcome variables as detailed in subsequent sections, to identify any baseline predictors of responsiveness. Two different randomisation lists, independent of each other, will, therefore, be used. One randomisation list will determine the order of placebo air versus oxygen-filled canisters for the placebo-controlled 6MWT occurring at the baseline visit, the other randomisation list will determine the order of oxygen versus no intervention for the 4-week trial.

Following completion of the 2 weeks on the treatment arm assigned during the baseline visit (ambulatory oxygen or no intervention), patients will cross over to receive the alternative treatment for a further 2 weeks. The study design is outlined in Additional file 2: Figure S1. More details are available in the AmbOx protocol, version 3.0, dated 1 December 2014 (Additional file 1).

### Location and setting

AmbOx is sponsored by the Royal Brompton and Harefield NHS Foundation Trust and will recruit subjects from three UK centres (RBH; Aintree University Hospital led by LGS; Bristol ILD Unit led by HA), all with expertise in ILD.

### Study population and eligibility criteria

A total of 80 subjects will be enrolled and randomised. Subjects should fulfil the following criteria:

- A diagnosis of fibrotic lung disease, including idiopathic pulmonary fibrosis, fibrotic nonspecific interstitial pneumonia, fibrotic hypersensitivity pneumonitis, fibrotic organising pneumonia and nonclassifiable fibrotic ILD. Patients with fibrotic sarcoidosis or connective tissue disease (CTD)-associated fibrotic ILD will be included provided that there is no significant musculoskeletal involvement
- Patients whose oxygen saturation (SaO_2_) at rest on room air is ≥94% and falls to ≤88% on a baseline 6MWT
- Patients with stable symptoms (no changes in medications and no chest infections) and treatment during the period of 4 weeks prior to being randomised into the study, including the 2-week run-in period
- Patients able to provide written informed consent

The Patient Information Sheet and Informed Consent Form can be found in Additional files 3 and 4, respectively. Subjects should not enter the study if any of the exclusion criteria listed in Additional file 5 are present. Patient will have had an echocardiogram and spirometry performed within 6 months prior to the screening visit or within 6 weeks following this. Serum brain natriuretic peptide (BNP) levels will be collected for all patients within a month from the screening visit.

### Intervention

Light-weight oxygen gas cylinders will be used to provide oxygen during activities (ambulatory oxygen). Ambulatory oxygen will be provided by the relevant oxygen companies in the UK, through submission of a Home Oxygen Order Form (HOOF) based on the location of the patient participating in the study. Overall, there are four oxygen companies which provide supplemental oxygen in the UK, including Air Liquide (which services North and South London, North and South West England, and the East Midlands), BOC (Eastern England and NorthEast), Baywater Healthcare (Yorkshire and Humberside, West Midlands and Wales) and Dolby Vivisol (South Central England and South East Coast). Continuous oxygen flow via nasal cannulae will be used for all patients to standardise mode of delivery. Oxygen flow rates will be determined on the basis of the entry 6MWT on oxygen, so as to maintain oxygen saturation of ≥90% for at least half of the 6MWT, where possible. If the patients are randomised to start on oxygen on the first 2 weeks of the 4-week trial period, the company will be asked to remove any unused oxygen cylinders on the day of the crossover to the 2 weeks ‘off’ oxygen. To determine oxygen use during the 2 weeks when patients are randomised to be on treatment, patients will be asked to keep a daily diary recording the number of hours spent using oxygen. Investigators will also ask the relevant oxygen company to provide the number of cylinders delivered to the patient per week along with the number of any unused or partially used cylinders (approximating to the nearest 25%) at the end of the 2 weeks on oxygen, wherever possible. To further validate patient-reported use of oxygen cylinders, a researcher at the Aintree site will sample 10 consecutive patients living in the nearby area and will visit patients at home at the end of the 2 weeks of ambulatory oxygen to independently assess numbers of fully used, half-used and full oxygen cylinders. Comparison between patient- and researcher-reported cylinders use will be included in data analysis. More information is provided in Section 13 of the study protocol.

The study design does not include a placebo arm because:

- The intervention is a combination of possible benefits from oxygen and the disadvantage of canister weight. These cannot be separated. Placebo control is impossible because there is no means of providing placebo weight. Attempts to control solely for oxygen use without taking canister weight into account would not be clinically meaningful. In a recent COPD study, cylinder weight was reported as a barrier to use by 93% of study participants [22]
- A positive result against an air-filled canister arm would be clinically uninterpretable. This is a study design in which the ‘placebo’ would be actively harmful to study participants. Carriage of an air-filled cylinder would be expected to lead to earlier desaturation and reduced exercise tolerance. Such a design would not inform the real-life comparison between oxygen plus cylinder and no intervention
- It should also be stressed that objective measures of change will be evaluated as secondary endpoints: we expect to explore correlations between these variables and the primary endpoint to exclude the possibility that an observed treatment benefit on the primary endpoint might be confounded by a placebo effect

### Concomitant medication

It will be possible to introduce any rescue medication during the study period as judged appropriate by the treating physician. However, the aim is to enrol subjects whose treatment is not expected to change during the 4-week treatment period of the trial. Patients who are on established treatment for their ILD, with immunosuppressants, corticosteroids, N-Acetyl Cysteine (NAC) and/or antifibrotic agents, will continue on their regular medication. Concomitant medication will be recorded in patient’s notes and the Case Report Forms (CRFs).

## Outcomes

### Primary outcome measure

- Difference in the health status measured by the K-BILD (global score and its three domains) [16] between the two treatment arms (ambulatory oxygen versus no oxygen) at the end of the relevant treatment period

### Key secondary outcome measures

- Difference in the UCSD Shortness of Breath Questionnaire at the end of each treatment period (oxygen versus no oxygen) [17]
- Difference in global patient assessment scores [18]

### Additional secondary outcome measures

- Difference in health-related quality of life scores (SGRQ, HDAS) [19]
- Difference in activity parameter scores (SenseWear Armbands)
- Difference in patient-reported activity levels as per patient daily diaries
- Difference in the 48-h oxygen saturation scores recorded by portable oximeters (to be also used as an indirect measure of compliance)
- Difference in the 48-h oximeter recorded heart rate
- Analyses on the placebo-controlled 6MWT on oxygen versus placebo air cylinders to assess whether the response to oxygen on the 6MWT predicts benefit of ambulatory oxygen in day-to-day life
- Oxygen cylinder use
- Safety and tolerability

### Explanatory outcome measures

- Benefit of ambulatory oxygen in relation to the following:
  - o Improvement in baseline 6MWT performance on oxygen (at the flow determined during the screening visit) compared to placebo air
  - o Outcome in relation to underlying ILD diagnosis: IPF versus non-IPF
  - o Outcome in relation to the presence of pulmonary vascular disease/pulmonary hypertension (PH), defined on the basis of baseline echocardiogram (right ventricular systolic pressure (RVSP) >40 mmHg and/or right ventricular (RV) dilatation/reduced function) and/or elevated baseline serum BNP levels

### Qualitative interviews

The use of semistructured interviews in a subset of patients at the end of the study is aimed at identifying patient-derived expectations and concerns over the psychological and practical barriers faced when using ambulatory oxygen.

### Participant timeline

**Fig. 1:**
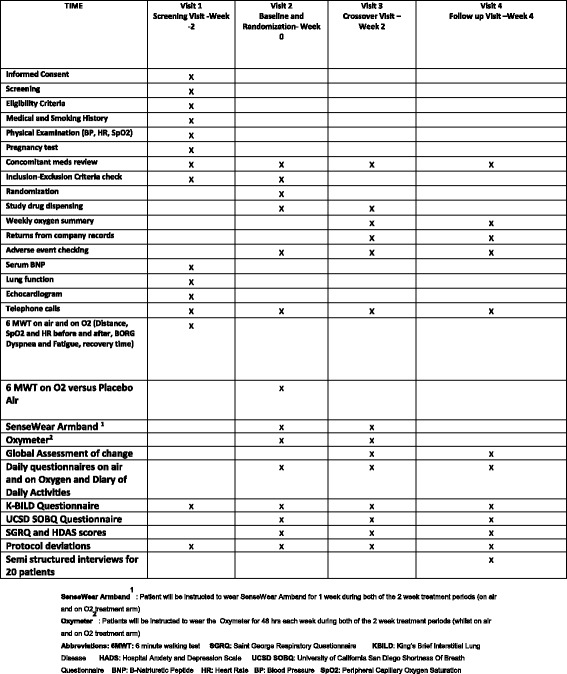
SPIRIT Schedule of Events

The time schedule for the enrolment, interventions and assessments for participants is shown in Additional file 2: Figure S1 and in Fig. 1. An indexed Standard Protocol Items: Recommendations for Interventional Trials (SPIRIT) Checklist can be found in Additional file 6.

### Assignment of interventions

Randomisation allocation as to the order of treatment will be released using an interactive web-based randomisation system (InForm), set up by Imperial College Clinical Trials Unit (ICTU). Simple, block-method randomisation will be performed to determine the order of treatment.

All study patients will be randomised during baseline visit as follows:

- Randomisation as to the order in which subjects will perform the 6MWT on oxygen versus air. The patients will be blind to the content of the canisters
- Randomisation as to the order in which subjects will have the portable home oxygen versus no oxygen during the first 2 weeks of treatment

### Sample size estimate

As stated above, the primary outcome measure is the difference between the health status score measured by the K-BILD collected after 2 weeks on ambulatory oxygen and the K-BILD collected after 2 weeks on no oxygen. The repeatability of the K-BILD at 2 weeks in patients with stable ILD is high, with an intraclass coefficient for domains and total score of 0.86–0.94 [16]. This study is powered to detect a difference of 8 units in the K-BILD total score, the minimally clinically important difference (SD, 20), recently estimated [23].

We calculated that a sample size of 80 randomised patients would allow 90% power at 5% significance to detect a difference of at least 8 points between the two crossover trial arms, allowing for a 15% dropout rate, in view of the high morbidity/mortality of the disease. This degree of power will also allow us to perform some explanatory subgroup analyses, as out lined above. Although the study is powered to allow a 15% dropout rate, we will make every effort to minimise patient loss and missing data during the trial period [24]. In order to maximise completeness of data, patients will be called at the end of each week during the trial period. They will be reminded to wear the activity monitor and/or the oximeter for the allocated time, and will be encouraged to continue filling in their daily activity diaries. The software used for power calculations was ‘PS Power and Sample Size calculator version 3.0 by William Dupont and Walton Plummer 2009’. For the qualitative component, it is anticipated that a subsample of *N* = 20 will be adequate to achieve theoretical saturation.

### Data collection and management

#### Quality of life/Shortness of Breath Questionnaire data

The primary outcome measurement will be the difference in K-BILD scores on completion of the 2 weeks on ambulatory oxygen compared to no oxygen. All questionnaires will be self-administered during the study visits, and will be completed at baseline and repeated at the follow-up crossover visit after 2 weeks since the baseline visit and at the final follow-up visit at 4 weeks. In addition to the K-BILD, patients will be asked to complete the UCSD SOBQ [17], a widely validated index, designed to assess activity-related dyspnoea, as well as the global patient assessment of change [18], the SGRQ [19] and the HADS, both validated in ILD [20].

#### Activity assessment

During the second week of each of the 2-week periods of the study treatment, patients will be asked to wear an activity monitor during waking hours, the SenseWear Armband (Bodymedia – Pittsburgh, PA, USA), which measures energy expenditure, daily number of steps, and time spent at different levels of physical activity. SenseWear-derived measurements are sensitive and repeatable in patients with chronic lung disease [25, 26]. Patients will also be asked to complete a diary of daily activities as measure of physical activity by using a custom designed diary modelled on a modified diary method of Follick et al. [27, 28]. Participants will be asked to complete this diary at least three times a day, recording the activity undertaken for the majority of each 2-h block, in addition to specifying whether oxygen cylinders were used. In addition, total hours of outings each day will be recorded.

#### Oxygen saturation

For 48 h during the second week of each treatment period, patients will also be asked to wear a portable oximeter to assess oxygen saturation for 48 continuous hours. Measures of continuous oxygen saturation will also allow control for self-reported use of ambulatory oxygen.

At the end of each 2-week treatment period, the SenseWear Armbands, the oximeters and the patient diaries will be collected.

### Assessment of compliance of oxygen cylinder use

Oxygen use will be expressed as number of full, half-full and unused cylinders as self-reported by the patient after each of the 2 weeks on oxygen. Patient-reported use will be cross-checked by assessing accountability records provided by the relevant oxygen company, following an agreement that has been put in place.

To validate patient-reported use of oxygen cylinders, a researcher at the Aintree site will sample 10 consecutive patients living in the nearby area. The researcher will visit patients at home at the end of the 2 weeks of ambulatory oxygen to independently assess numbers of fully used, half-used and full oxygen cylinders. Comparison between patient- and researcher-reported cylinders use will be included in data analysis.

Patients will also be asked to fill in a daily oxygen use diary card to write down the time of use of oxygen canisters, and the activity being performed. This will be cross-checked with the continuous oxygen saturation data recorded by the portable oximeter, which patients will be asked to wear for 2 days a week, so as to correlate the two.

### Qualitative assessments

The impact of using ambulatory oxygen will be identified through qualitative semistructured interviews with a purposive sample of 20 patients. The interviews will explore patients’ and their informal carers’ perspectives on how the ambulatory oxygen has affected their day to day life. The interview will be conducted within 2 weeks of the end of treatment visit. Patients from the RBH site will be approached by a qualitative researcher at the end of treatment visit and asked if they will participate in the qualitative interview (as described in the Patient Information Sheet – Additional file 4). Consent of the patient and carer will be confirmed before proceeding to the interviews at a venue convenient to the patient.

The audio-recorded, semistructured interviews will use a topic guide focussing on practical barriers to optimal oxygen usage, practical, social and psychological difficulties encountered, concerns about dependency, and views on the information required prior to ambulatory oxygen prescription. The interviews will also explore patients’ experience of participating in the trial. Field notes will be written after each interview to aid reflexive analytical processes [29]. These interviews will also allow patients to provide suggestions on how to improve the provision and future design of ambulatory oxygen provision and devices as well as their views on the trial design.

### Safety assessments

Checking for the occurrence of adverse events and clinical endpoints will begin from randomisation and will continue until study completion. At each study visit the investigator or designee will make an assessment of safety and will specifically review the clinical history and investigation findings with regard to the occurrence of adverse events (AEs) or serious adverse events (SAEs). Details of adverse and clinical events will be captured on the trial electronic Case Report Form (eCRF).

### Blood samples

Blood samples will be taken at the start of the 4-week period to evaluate serum BNP levels. All BNP measurements will be performed in the same laboratory at the RBH.

### Health economics

The AmbOx study will include an economic evaluation to assess the cost-effectiveness of ambulatory oxygen use in patients diagnosed with fibrotic ILD compared to no ambulatory oxygen, from the perspective of the NHS. As ambulatory oxygen is not anticipated to have any effect on disease progression in the short term, costs related to unplanned health professional appointments or hospital admissions will not be included. The primary measure of benefit for the economic evaluation will be change in K-BILD score. Quality of life benefits will also be assessed using quality-adjusted life years (QALYs). QALYs will be estimated by synthesising utility values from existing medical literature for health states described by the K-BILD or SGRQ. Discounting will not be applied given the short trial duration. Cost-effectiveness will be expressed as the incremental cost per unit improvement in K-BILD and per QALY gained.

We expect that ambulatory oxygen will allow patients to lead an active and independent life for longer, with a varied set of longer-term benefits, reducing the period of dependency and expensive care packages associated with the final stages of fibrotic ILD, characterised by severely limited mobility. Therefore, future research should consider modelling the longer-term costs and benefits of ambulatory oxygen when sufficient data are available to evidence this potential benefit. The economic evaluation conducted alongside this trial will provide important preliminary guidance on the likely cost-effectiveness of implementing ambulatory oxygen in fibrotic ILD, as well as evidence to support future studies that extrapolate findings to a longer time horizon.

### Discontinuation or withdrawal of study subjects

We do not expect there to be any significant toxicity from the use of ambulatory oxygen. Subjects will be withdrawn from the trial only if they wish to do so, or if they develop an unexpected worsening in their condition which means that they require oxygen at rest. Patients withdrawing from the study will be asked, if willing and whenever possible, to fill in the relevant questionnaires at the end of the 2- and 4-week period, if needed via telephone, so as to be able to compare change in health status and other questionnaire-related outcomes, compared to baseline, against subjects completing the trial.

### Adverse event (AE) and serious adverse event (SAE) reporting

AEs and SAEs will be identified according to standard criteria and will be recorded in the eCRF and reported to the sponsor. Given the nature of the participants’ underlying disease, expected AEs and SAEs include disease progression, lower respiratory tract infections, hospitalisation, and death.

### Data management and data checking

Data will be collected on an eCRF system. The InForm system will be used to develop the eCRF and will be designed in accordance with the requirements of the clinical trial protocol and will comply with regulatory requirements. Local personnel will be trained on the InForm system. Access will be restricted to site personnel, trial managers, trial monitors and the data management team. Personnel will have individual log-on and passwords. It will be the investigator’s responsibility to ensure the accuracy of all data entered and recorded in the eCRFs. Trial monitors will check the accuracy of the eCRF data against source documents. It is anticipated that the majority of source data (medical progress notes and letters, tests and investigations) will be filed in the individual patients’ medical records. Any deviation from source data being present in the medical notes will be identified and documented. The eCRF and source documents must be available at all times for review by the sponsor’s clinical trial monitor, auditors and for inspection by the Medicines Health Regulatory Agency. The accuracy of eCRF data will be verified by review of the source documents and details will be provided in the Trial Monitoring Report.

### Statistical analysis

Before starting the data analysis, the level and pattern of the missing data in the baseline variables and outcomes will be established by forming appropriate tables. The likely causes of any missing data will be investigated. This information will be used to determine whether the level and type of missing data have the potential to introduce bias into the analysis results for the proposed statistical methods, or to substantially reduce the precision of estimates related to treatment effects.

The potential effects of performance, attrition and evaluation bias associated with the lack of a placebo arm will be considered. Although we are not able to fully exclude bias in the patient-reported outcomes, this will be minimised by exploring correlations between the primary endpoint and secondary outcomes, including analysis of SenseWear Armband activity monitors, to assess the possibility that a placebo effect is associated with evaluation bias. Attrition bias should be minimised by the fact that there will be no change in dosing for the duration of the trial, and we do not expect there to be any toxicity from the use of ambulatory oxygen. Subjects will be withdrawn from the trial only if they wish to do so or because of disease progression and the development of hypoxia at rest. We do not expect there to be any bias in the background provision of care (performance bias) depending on use or not of oxygen, as patients enrolled into the trial will be those not expected to change their treatment for the duration of the study.

Although we will not adjust for baseline K-BILD score in the primary analysis, secondary analyses using the K-BILD filled in at baseline visit (week 0) as a covariate, or mean of the K-BILD score at screening visit (week −2) and at baseline visit (week 0) will be considered.

### Primary outcome measure

- The primary outcome will be the difference in K-BILD score on oxygen compared to no oxygen. We will use a generalised linear model with the difference in health status as the independent variable and treatment sequence as a covariate. If the assumption of the mixed model is not met, data will be either log-transformed and/or other nonparametric methods will be considered. Any adjusted analyses will be performed as secondary sensitivity analysis including adjusting for any covariate that is itself linked to the primary outcome (see below)
- Analysis of the primary outcome will be by intention-to-treat. The data will be analysed according to the initial randomisation groups irrespective of subsequent withdrawals. However, should there be a sizeable frequency of protocol variation, per protocol analysis will also be performed
- The hypothesis to be tested is that ambulatory oxygen improves health status compared to no treatment. The study will be considered positive if statistical significance at the level of 0.05 (two-tailed) is achieved
  - o Sensitivity analyses will include adjustment for any covariates linked to the primary outcome. These may include baseline K-BILD measurement, age, gender, smoking history, Body Mass Index (BMI), ILD severity as measured by the Composite Physiologic Index (CPI), presence of PH as measured by echo, baseline BNP levels, etc.
  - o Patient-reported consumption of oxygen cylinders will also be factored in as a covariate. Its validity will be checked against oxygen company-reported cylinder consumption wherever possible, and, in a subgroup of patients, by direct observation by clinical nurse specialist (CNS) home visits (Aintree centre)
  - o To assess the relationship between the response to supplemental oxygen measured with a series of parameters during the placebo air versus oxygen 6MWT and the benefit seen in day-to-day life with ambulatory oxygen, to identify any baseline predictors of responsiveness. These parameters will be included in the statistical model as fixed- effect variables. Possible 6MWT predictors to be analysed will include area under the curve of oxygen saturation, walked distance, maximum/minimum and mean heart rate, end-test dyspnoea, end-test oxygen saturation, end-test heart rate, and recovery times to baseline dyspnoea, heart rate and saturation
  - o Exploratory sensitivity analyses of the effects of ambulatory oxygen will be conducted in predefined subgroups, including:
    - ▪ IPF versus non-IPF
    - ▪ Patients with and without evidence of PH
    - ▪ Patients stopping and not stopping during the baseline 6MWT on air
- STATA software will be used for statistical analysis

### Secondary outcomes

- Comparison of differences in secondary outcome measures on and off ambulatory oxygen treatment, will be performed by similar methods as for the primary outcome

### Qualitative interview analysis

Interviews will be transcribed verbatim, checked and anonymised. Transcripts will be analysed thematically using a framework approach [30]. NVivo 10 software (QSR International Pty Ltd., www.qsrinternational.com) will be used to manage and index the data prior to charting, mapping and interpretation. Framework analysis provides a systematic approach to analysis useful for applied qualitative research and enhances visibility of analysis for policy-makers and practitioners. Further, it is particularly useful for linking qualitative to quantitative data. This would facilitate a mixed-methods analysis of the broader dataset, thus providing comprehensiveness and greater knowledge yield [31, 32], making best use of the data collected.

### Patient public involvement: patient/carer focus group

Patients and carers living with ILD who are not participating to the study will be invited to participate to a focus group to provide patient and carer perspectives on a sample of the anonymised interview transcripts and their interpretation of the analysis.

### Interim analyses

There will be no formal interim analysis as the study duration is short, and we do not expect any significant undesirable outcomes. A regular review of safety data will be conducted to monitor the safety of patients in the trial. A Data Monitoring Committee (DMC) will follow number of deaths, early discontinuation due to AEs and SAEs in an unblinded fashion. A planned DMC meeting will be held to review all available data twice yearly.

### End of study

The final study visit will be the one at the end of the second of the 2-week study periods The trial will formally end when the final subject completes their week-4 visit.

Data from the study will be published in abstract form at international meetings and will be submitted for publication in a peer-reviewed journal and will be reported to the study funder.

## Discussion

Fibrotic lung diseases are an important cause of morbidity and mortality, with a devastating impact on quality of life. With currently available treatments providing only partial effectiveness and often, at best, stability of disease, interventions to improve day-to-day quality of life are crucial in this group of patients. This trial will be the first to prospectively assess the impact of ambulatory oxygen in patients with fibrotic ILD and to explore the outcomes for which it is most effective, the predictors of benefit and the variables/patient characteristics associated with lack of response. This study will form an essential precursor to the development of ILD-specific guidelines on ambulatory oxygen use. Ultimately, if proven to be effective and cost-effective, the appropriate prescription and use of ambulatory oxygen should lead to prolonged patient independence and mobility, allowing maintenance of fitness levels and of active roles in daily life, with increased psychological and physical wellbeing, as well as potentially reduced health-related costs.

### Trial status

Recruitment to AmbOx began in August 2014. The trial is currently actively recruiting in the UK.

## Additional files

Additional file 1: AmbOx protocol v3.0. (PDF 440 kb)

Additional file 2: Figure S1. Flow diagram. (PPTX 269 kb)

Additional file 3: Patient Information Sheet: Version 3.0, 1 December 2014. (DOC 337 kb)

Additional file 4: Informed Consent Form: Version 3.0, 1 December 2014. (DOC 32 kb)

Additional file 5: Exclusion criteria. (DOCX 15 kb)

Additional file 6: SPIRIT Checklist. (DOC 123 kb)

## Abbreviations

ILD: Interstitial lung disease
K-BILD: King’s Brief Interstitial Lung Disease Questionnaire
SaO_2_: oxygen saturation
SGRQ: St. George’s Respiratory Questionnaire
UCSD SOBQ: San Diego Shortness of Breath Questionnaire.
