# Supplementary material for: Ambulatory oxygen in fibrotic lung disease (AmbOx): study protocol for a randomised controlled trial
Source: Trials. 2017 Apr 28;18:201. doi: 10.1186/s13063-017-1912-9 (PMC5410093; doi:10.1186/s13063-017-1912-9)
Supplement: Supplementary file 1 — AmbOx protocol v3.0. (PDF 440 kb) [file 13063_2017_1912_MOESM1_ESM.pdf]

STUDY PROTOCOL

Randomised controlled, crossover trial to evaluate the Effects of Ambulatory Oxygen on health status in patients with Fibrotic Lung Disease (FLD)

Sponsor's R&D Registration Number: 2013OE005B
Sponsor's Details: Royal Brompton and Harefield NHS Foundation Trust (RB&HFT)
EudraCT number: 2013-004355-20

CHIEF INVESTIGATOR (CI):
Dr Elisabetta Renzoni
Consultant Chest Physician
Royal Brompton Hospital (RBH)
Fulham Wing, Level M

London SW3 6LR

Phone: 0207 3528121 ext 3367 or 8327
Email: e.renzoni@rbht.nhs.uk or e.renzoni@imperial.ac.uk
Fax: 0207 3518336

SPONSOR REPRESENTATIVE:

Dr Angela Cooper
Royal Brompton and Harefield NHS Foundation Trust (RB&HFT)
Royal Brompton Hospital (RBH)
Research Office
Chelsea Wing, Level 2
Sydney Street
London SW3 6NP

Phone: 0207 352 8121 ext. 2610
Email: a.cooper@rbht.nhs.uk
Fax: 020 8725 0794

Information in this protocol is confidential and should not be disclosed, other than to those directly involved in the execution or the ethical/regulatory review of the study, without written authorization from RB&HFT Research Office (RO) or its affiliates.

Signature Page and Statement

The Chief Investigator (CI) and the RO have discussed this protocol. The investigators agree to perform the investigations and to abide by this protocol except in the case of medical emergency (Section 10.10) or where departures from it may be mutually agreed in writing.

The Investigator agrees to conduct the trial in compliance with the protocol, GCP and UK Regulations for CTIMPs, the Data Protection Act (1998), the Trust Information Governance Policy (or other local equivalent), the Research Governance Framework (2005), the Sponsor's SOPs, and other regulatory requirements as appropriate.

This protocol has been written in accordance to the Sponsor's procedure for writing study protocols outlining study procedures for the conduct and management of Clinical Trials of Investigational Medicinal Products (CTIMPs).

**Chief Investigator (CI)**		
Elisabetta Renzoni<br>Consultant Chest Physician		
Royal Brompton and Harefield NHS Foundation Trust (RB&HFT)	Signature	Date
**Sponsor Representative**		
Dr Angela Cooper<br>Associate Director of Research		
Royal Brompton and Harefield NHS Foundation Trust (RB&HFT)	Signature	Date

Contents

Signature Page and Statement 2
Contents 3
1 List of abbreviations 6
2 Study personnel 8
3 Study synopsis 9
4 Introduction 11
4.1 Background 11
4.2 Investigational Medicinal Products (IMPs) 11
4.3 Pre-clinical data 11
4.4 Clinical data 11
4.5 Study Rationale and risk/benefit analysis 12
4.6 Management of potential study risks 13
5 Study objectives 13
5.1 Primary objective 13
5.2 Secondary objective 14
6 Trial design 14
6.1 Overall design 14
6.1.1 Target population and screening: 15
6.1.2 Two weeks Run in period 15
6.1.3 Baseline Assessments – Visit 2 in Week 0 15
6.1.4 Ambulatory Oxygen crossover Trial procedure 16
6.1.5 Ambulatory Oxygen Unit 16
6.2 Dosage regimen and rationale 16
6.3 Concomitant treatment 16
6.4 Schematic of trial design 17
7 Eligibility criteria 17
7.1 Inclusion criteria 17
7.2 Exclusion criteria 18
8 Subject/Patient Recruitment process 18
9 Study procedures 19
9.1 Informed Consent procedure 19
9.2 Randomisation procedure 20
9.3 Emergency un-blinding 20
10 Study Assessments 20
10.1 Timing of Assessments 20
10.2 Screening assessments at Visit 1 (Week -2) 20
10.3 Two weeks ‘run in period’ 22
10.4 Baseline assessments and Randomization (Visit 2 in Week 0) 22
10.5 Crossover Visit – Visit 3 in Week 2 23
10.6 End of Treatment Visit – Visit 4 in Week 4 24
10.7 Qualitative assessments 24

10.8 Unscheduled Visits 25
10.9 Summary of study Assessments 25
10.10 Summary flow chart of study assessments 26
11 Methods 26
11.1 Laboratory procedures 26
11.2 Radiology or any other procedure(s) 26
11.3 Definition of the End of Trial 27
12 Discontinuation/withdrawal of participants and stopping rules 27
13 IMPs and non-IMPs used in the trial 27
13.1 Name and description of each IMP 27
13.2 Accountability procedure for the IMPs 28
13.3 Route of administration, dosage, dosage regimen, and treatment period(s) of the IMPs 29
13.4 Dosage modifications 29
13.5 Assessment of compliance 29
13.6 Post-trial IMP arrangements 29
13.7 Name and description of each non-IMP (NIMP) 29
14 Pharmacovigilance 29
14.1 Definitions 30
14.2 Terminology for classification of SAEs 30
14.3 Recording of Safety Information 31
14.3.1 Adverse Events (AEs) 31
14.3.2 Serious Adverse Events (SAEs) 32
14.3.3 Specific Adverse Events to be captured 32
14.3.4 Expected SAE/Rs related to IMP 32
14.3.5 Expected SAEs related to underlying disease 32
14.4 Reporting Adverse Events (AEs) – (including SAE/Rs) 33
14.4.1 SUSARs 33
14.5 Notification of deaths 33
14.6 Reporting to Regulatory Authority 33
14.7 The type and duration of the follow-up of subjects after AEs 34
14.8 Development Safety Update Reports 34
14.9 Annual Progress Reports (APRs) 34
14.10 Pregnancy 34
14.11 Reporting Urgent Safety Measures 34
14.12 Notification of Serious Breaches of GCP and/or the protocol 35
15 Data management and quality assurance 35
15.1 Confidentiality 35
15.2 Source Documents 35
15.3 Data collection tool 36
15.4 Data handling and analysis 37
16 Archiving arrangements 37

17 Statistical design 37
17.1 Statistical input in trial design 37
17.2 Endpoints 37
17.2.1 Primary endpoints 37
17.2.2 Secondary endpoints 38
17.3 Sample size and recruitment 38
17.3.1 Sample size calculation 38
17.3.2 Planned recruitment rate 39
17.4 Statistical analysis plan 39
17.4.1 Summary of baseline data and flow of patients 39
17.4.2 Primary endpoint analysis 39
17.4.3 Secondary endpoint analysis 40
17.5 Randomisation 40
17.6 Interim analysis 40
18 Committees in involved in the trial 40
19 Direct access to source data 41
20 Ethics and regulatory requirements 41
21 Monitoring plan for the trial 41
22 Finance 42
23 Insurance and indemnity 42
24 Publication policy 42
25 Statement of compliance 42
26 References 43
27 Appendices 46
27.1 Appendix 1 46

1 List of abbreviations

AE	Adverse Event
AR	Adverse Reaction
ASR	Annual Safety Report
BNP	B-natriuretic peptide
CA	Competent Authority
CI	Chief Investigator
CRF	Case Report Form
CRO	Contract Research Organisation
CTA	Clinical Trial Authorisation
CTIMP	Clinical Trial of Investigational Medicinal Product
DMC	Data Monitoring Committee
EC	European Commission
EMA	European Medicines Agency
EU	European Union
EUCTD	European Clinical Trials Directive
EudraCT	European Clinical Trials Database
EudraVIGILANCE	European database for Pharmacovigilance
EXACT	Exacerbations of Chronic Pulmonary Disease Tool
GAfREC	Governance Arrangements for NHS Research Ethics
GCP	Good Clinical Practice
GMP	Good Manufacturing Practice
IB	Investigator Brochure
ICF	Informed Consent Form
IMP	Investigational Medicinal Product
IMPD	Investigational Medicinal Product Dossier
ISF	Investigator Site File
ISRCTN	International Standard Randomised
MA	Marketing Authorisation
Main REC	Main Research Ethics Committee
MHRA	Medicines and Healthcare products Regulatory Agency
MRC	Medical Research Council
MS	Member State
MUST	Malnutrition Universal Screening Tool
NHS R&D	National Health Service Research & Development
NIMP	Non- Investigational Medicinal Product
PI	Principal Investigator
PIS	Participant Information Sheet
QA	Quality Assurance
QC	Quality Control
QP	Qualified Person for release of trial drug
RCT	Randomised Control Trial
REC	Research Ethics Committee
SAR	Serious Adverse Reaction

SAE	Serious Adverse Event
SDV	Source Document Verification
SOP	Standard Operating Procedure
SmPC	Summary of Product Characteristics
SSA	Site Specific Assessment
SSAR	Suspected Serious Adverse Reaction
SUSAR	Suspected Unexpected Serious Adverse Reaction
TMG	Trial Management Group
TSC	Trial Steering Committee

2 Study personnel

Principal Investigator (PI): Dr Elisabetta Renzoni
E-mail: e.renzoni@rbht.nhs.uk
Phone: 0207 352 8121 ext 3367 8327
Fax: 0207 351 8336

Statistician: Winston Banya
E-mail: w.banja@rbht.nhs.uk
Phone: 0207 3528121 ext 2707

Medical Expert: Prof Athol Wells
E-mail: a.wells@rbht.nhs.uk
Phone: 0207 3528121 ext or 8327
Fax: 0207 351 8336

Pharmacy: Royal Brompton and Harefield NHS Foundation Trust
E-mail: j.foy@rbht.nhs.uk
Phone: 0207 352 8121 Ext. 4080/5419
Fax: 0207 351 8904

Co-Investigators: Dr Lisa Spencer,
Email: LISA.SPENCER@aintree.nhs.uk
Phone: 0151 529 2479

Dr Surinder Birring
Email: surinder.birring@nhs.net

Dr Sharon Fleming,
Email: s.fleming@rbht.nhs.uk
Phone: 0207 351 8845

Dr Nicholas Hopkinson
Email: n.hopkinson@ic.ac.uk
Phone: 020 7349 7775

Dr Morag Farquhar
Email: mcf22@medschl.cam.ac.uk
Phone: 0122 376 9294

Professor Paul Cullinan,
Email: p.cullinan@imperial.ac.uk
Phone: 0207 351 8934

Dr John Tayu Lee,
Email: t.lee@imperial.ac.uk

3 Study synopsis

**Full study title:**	Randomized, controlled crossover trial to evaluate the effects of Ambulatory Oxygen on health status in patients with Fibrotic Lung Disease (FLD).
**Short study title:**	Ambulatory Oxygen in Fibrotic Lung Disease (FLD)
**Study R&D number:**	2013OE0058B
**Study drug:**	Medical Oxygen
**Chief Investigator:**	Elisabetta Renzoni
**Study centres/sites:**	1. Royal Brompton Hospital ILDU<br>2. Respiratory Medicine Aintree Hospital<br>3. Respiratory Medicine King's College Hospital (KCL)
**Study duration:**	30 months
**Clinical phase:**	Phase IV
**Primary Objective:**	• Change in health status (as assessed by K-BILD questionnaire)
**Secondary Objective:**	• Dyspnoea scores, global patient assessment, monitored and patient-recorded activity parameters, Quality of Life (QoL) scores.<br><br>• In a subgroup of patients, semi-structured interviews at the end of the four-week period will be conducted to evaluate patients' and carers' experiences with ambulatory oxygen and trial participation.
**Study population:**	Patients with Fibrotic Lung Disease (FLD), including the idiopathic interstitial pneumonias and fibrotic hypersensitivity pneumonitis.
**Methodology:**	Four-week randomized crossover trial of ambulatory oxygen for use during physical activity.
**Eligibility criteria:**	*Inclusion criteria:*<br>1. IPF or any other Fibrotic Lung Disease (FLD)<br>2. patients aged 18 – 99 yrs<br>3. Desaturation ≤ 88% on a 6MWT on room air<br>4. Stable respiratory symptoms (no changes in medications and no chest infections) in the 4 weeks preceding the randomisation including the run in period
	Exclusion criteria:<br>1. Patients meeting criteria for long term oxygen therapy, SaO_2 at rest on room air < 94%<br>2. Patients expected to change treatment during the course of the study<br>3. Significant locomotor or communication difficulties<br>4. Patients with sarcoidosis or connective tissue disease

	affecting the musculoskeletal system<br>5. Current smokers<br>6. Pregnancy<br>7. History of symptomatic ischaemic cardiac disease (exertion-induced chest pain)<br>8. Anaemia, Hb < 9g/dl
**Study drugs, Dose and Mode of Administration:**	
Medical oxygen (Oxygen Ph. Eur. 100%) PL 15929/0005, obtained through the Home Oxygen Order Form (HOOF), supplied by Air Liquide (for the areas of South/North London, North West, East Midlands and South West), or by BOC (for the areas of Eastern England, North East) or by Dolby Vivisol (for the areas of South Central, South East Coast) or by Baywater Healthcare (for the areas of Yorkshire & Humberside, West Midlands, Wales) as per standard clinical practice. The light-weight portable oxygen gas cylinders will be used across sites to standardise mode of delivery.<br>The study will consist of a four week randomized crossover trial of ambulatory oxygen for use during physical activity. Patients meeting inclusion criteria and deemed clinically stable at the end of the run in period, will be assigned in random order to two weeks on ambulatory oxygen or not. Oxygen flow rates will be determined on the basis of the entry 6MWT on O_2, so as to maintain oxygen saturation ≥ 90% for at least half of the 6MWT duration, where possible. Modification of the flow rate is not anticipated for the duration of the study.	
**Duration of Treatment:**	Two weeks
**Criteria for evaluation:**	*Primary outcome variable:*<br>• change in the total “health status” score as assessed by the K-BILD questionnaire
	Secondary outcome variables:<br>• dyspnoea scores, global patient assessment, monitored and patient-recorded activity parameters, Quality of Life (QoL) scores.

4 Introduction

4.1 Background

Fibrotic Lung Diseases (FLDs) are chronic and progressive conditions resulting in substantial morbidity and mortality. The cardinal symptom of all fibrotic Interstitial Lung Diseases (ILDs) is shortness of breath. Although initially present only on strenuous activities, this unfortunately progresses until even routine activities of daily living become severely limited. This has a devastating impact on Quality of Life (QOL). Measures aimed at improving respiratory symptoms, social interactions and mobility are key to maintaining acceptable QOL standards for as long as possible. Patients with ILD can experience marked desaturation on exercise (1; 2). Exercise limitation in ILD is thought to be caused by multiple factors, including oxygen diffusion limitation, ventilation/perfusion mismatch and low mixed venous oxygen tension, although the pulmonary vasculature is the main limiting factor (3). In keeping with this, desaturation on exercise is significantly associated with pulmonary hypertension, and may contribute to pulmonary hypertension at rest (4). As pulmonary fibrosis advances, exertional breathlessness is triggered by ever decreasing activity levels. Eventually basic tasks, such as washing and dressing, become a challenge.

An improved understanding of the effects of ambulatory oxygen in patients with ILD should lead to significant benefits in terms of exercise performance, reduced symptoms of breathlessness and improved mobility in daily life. Currently, there is no standardised approach to the assessment and prescription of ambulatory oxygen in ILD patients. In many centres, the need for ambulatory oxygen in ILD is not routinely assessed. Although several centres including the centres participating to this study, routinely prescribe ambulatory oxygen to ILD patients with desaturation on exercise, there are no guidelines to direct ambulatory oxygen prescription, and no studies to indicate its benefits and drawbacks in patients with ILD.

4.2 Investigational Medicinal Products (IMPs)

Name of the study IMP: Medical Oxygen

Qualitative and Quantitative Composition: Oxygen Ph. Eur. 100%

Pharmaceutical Form: Inhalation Gas

Medical Oxygen used in this study is manufactured by Air Liquide Ltd., UK. Manufacturing Authorization, PL 15929/005 was issued on 04th February 1998 and renewed on 17th April 2003.

4.3 Pre-clinical data

There are very few studies investigating the physiological effects of supplemental oxygen on exercise capacity in ILD. Bye *et al* observed improvement in maximal oxygen uptake, maximal exercise workload and exercise duration in 16 patients with ILD breathing supplemental oxygen during incremental exercise (5).

4.4 Clinical data

We have recently completed a retrospective assessment showing that supplementary ambulatory oxygen improves the performance of a Six Minute Walk Test (6MWT) in patients with ILD (6). Frank and co-authors reported that further up titration of ambulatory oxygen in order to maintain saturation value above 90% or reach a 6L/min flow rate during 6MWT seemed to improve exercise capacity in IPF, as measured by a 6MWT, even in patients already using oxygen at home (7). Although the results of these retrospective reviews are highly encouraging, a prospective standardised study is needed to assess the impact of ambulatory oxygen on ILD patients' day to day Quality of Life (QoL).

4.5 Study Rationale and risk/benefit analysis

There are no prospective studies assessing whether ambulatory oxygen provides benefit to patients with ILD and no guidelines for use of oxygen in diffuse lung diseases. In particular, NICE guidelines for oxygen use only address COPD: (http://guidance.nice.org.uk/CG101), a disease which markedly differs from IPF or other ILDs, while the BTS document on oxygen use, only briefly touches on ambulatory oxygen (http://www.library.nhs.uk/GuidelinesFinder/ViewResource.aspx?resID=111561), and gives no specific guidance on its use in any of the Interstitial Lung Diseases (ILD). Specifically, there are no studies investigating the effects of ambulatory oxygen on day-to-day life in patients with Fibrotic Lung Disease (FLD), or assessing whether oxygen-induced improvements in 6MWT performance predict response to supplemental oxygen during activities of daily living, and an overall improvement in health status.

We have therefore planned a prospective study to assess whether ambulatory oxygen increases the health status of patients with Fibrotic Lung Disease (FLD) who experience oxygen desaturation on a 6MWT. The proposed project is the first of its kind in ILD, and addresses an under-researched area in urgent need of study. The study has the potential to lead to significant advances in the treatment and the understanding of exercise limitation in patients with ILD, and will represent an essential step towards the development of guidelines on oxygen use in ILD.

Currently, the prescription of ambulatory oxygen in ILD varies widely across the UK, with many respiratory centres only prescribing supplemental oxygen when the patient is hypoxic at rest. A 6MWT in ILD patients does not form part of the routine work up of ILD patients outside of specialised centres. This study aims to assess whether individuals with Fibrotic Lung Disease (FLD) whose SaO_2 falls ≤ 88% on a 6MWT benefit from the use of ambulatory oxygen in their daily lives, by assessing changes in health status.

The risks associated with the use of ambulatory oxygen are limited and highly manageable. As patients selected for the study will not be hypoxic at rest, and therefore will not be on long term oxygen, the risk of inappropriately low oxygen concentrations will not apply. The main reason for performing this study is to assess whether using ambulatory oxygen in ILD patients who desaturate on exercise, is associated with significant subjective benefits. One of the potential risks is that the lack of supplemental ambulatory oxygen during the two trial weeks off ambulatory oxygen could be associated with worse symptoms of breathlessness and increased fatigue. However, it is precisely because this is not yet known that the study is justified.

There is a theoretical risk of inappropriately high oxygen flows causing hypercapnia. However, this is mainly an issue in patients with COPD, where type II respiratory failure is relatively frequent. Conversely, in Fibrotic Lung Diseases (FLD), hypercapnia usually only occurs during the final stages of Fibrotic Lung Disease (FLD), when patients are hypoxic at rest and require continuous supplemental oxygen, a subgroup which will be excluded in this study.

Ambulatory oxygen will be provided through nasal cannula.

4.6 Management of potential study risks

In ILD patients who desaturate to ≤ 88% on a 6MWT, the physicians in the centres participating to this study (RBH, Aintree, King's) would be offering and prescribing ambulatory oxygen regardless of participation to the study, as part of local standard care. However, as stated previously, there are no national standards or guidelines to aid in the prescription of ambulatory oxygen in ILD patients in the UK. Many other centres do not routinely assess the need for ambulatory oxygen in lung fibrosis, and there has not yet been a study to show that it is effective. Therefore, patients will be asked to spend two of the four weeks not using ambulatory oxygen. This will not be different to what patients would have been doing just prior to the assessment (*i.e.* not using ambulatory oxygen). Although not using ambulatory oxygen could lead to increased breathlessness or reduced levels of activity compared to the two weeks on ambulatory oxygen, this is justified by the need for a study to assess the effectiveness of ambulatory oxygen.

The study team will contact patients by telephone during both the two weeks on oxygen and the two weeks on air. Should the patients struggle with breathlessness in the two weeks on air and request oxygen cylinders, this would be arranged and taken into account in the analysis of the study results. Patients who experience problems during the two weeks on air and require using oxygen will be asked to add this information to their patient diaries. The information will subsequently be recorded in the CRFs and the database.

Ambulatory oxygen is associated with a potential fire hazard. However, this is well known and routinely managed in the context of the existing ambulatory oxygen service. In this regard, all patients will be educated with regards to the potential fire hazards of supplemental oxygen, as routinely done by our oxygen services, as part of the standard prescription of ambulatory oxygen by our services. In view of the potential fire hazard, current smokers will be excluded.

There is a theoretical risk of hypercapnia, which is truly minimal, as this only occurs in a subgroup of patients during the terminal stages of fibrotic ILD, when hypoxia is present also at rest, and these patients will be excluded from the study.

5 Study objectives

5.1 Primary objective

The main aim of this project is to establish whether ambulatory oxygen in patients with fibrotic ILD whose oxygen saturation falls ≤ 88% on a 6MWT, leads to a significant improvement in their health status. The core of the project will be randomised, controlled

trial ambulatory oxygen used at home at an optimal flow rate determined by titration at screening visit and administered for a two-week period, compared to two weeks off oxygen.

5.2 Secondary objective

Secondary outcomes will include dyspnoea scores (including the University of California San Diego Shortness of Breath Questionnaire hereafter referred to as the Shortness of Breath Questionnaire), global patient assessment, monitored and patient-recorded activity parameters, and Quality of Life (QOL) scores assessed by the King's Brief Interstitial Lung Disease Questionnaire (K-BILD) and the St George's Respiratory Questionnaires, as well as the Hospital Anxiety and Depression Scale. We also aim to assess whether the improvement in 6MWT performance induced by portable oxygen can predict benefit of ambulatory oxygen in day to day living.

In addition, semi-structured interviews at the end of the four-week period in a subgroup of 20 patients will be conducted to evaluate patients' and carers' experiences regarding the use of ambulatory oxygen and trial participation.

6 Trial design

6.1 Overall design

The planned non-commercial study is a randomised, controlled crossover trial of ambulatory oxygen against no ambulatory oxygen over a four week period (two weeks on ambulatory oxygen and two weeks on air), to evaluate the effects of ambulatory oxygen on health status in patients with ILD. A short crossover study in this context has many advantages, since ambulatory oxygen has <u>immediate effect, with no wash-out period needed after use</u>.

At the start of the trial, the effects of ambulatory oxygen on 6 Minute Walk Test (6MWT) performances will be evaluated on oxygen and on air-filled canisters, with the patient blind to the contents of the canister, to assess whether oxygen-induced improvements in 6MWT parameters can predict its effectiveness in day to day life. The 6MWT is a well-established and highly reproducible test validated in ILD patients (8; 9), with significant prognostic implications (10).

The study design does not include a placebo arm because:

a) The intervention is a combination of possible benefits from oxygen and the disadvantage of canister weight. These cannot be separated. Placebo control is impossible because there is no means of providing placebo weight. Attempts to control solely for oxygen use without taking canister weight into account are clinically meaningless. In a recent COPD study, cylinder weight was reported as a barrier to use by 93% of study participants (11).

b) A positive result against an air-filled canister arm would be clinically uninterpretable. This is a study design in which the 'placebo' would be actively harmful to study participants. Carriage of an air-filled cylinder would be expected to lead to earlier desaturation and reduced exercise tolerance. Such a design would not inform the real life comparison between oxygen plus cylinder and no intervention.

c) All this aside, blinding is legally impossible (UK health and safety regulations **require** that oxygen cylinders for home use must be clearly labelled).

It should also be stressed that objective measures of change are evaluated as secondary end-points: we expect to explore correlations between these variables and the primary end-point to exclude the possibility that an observed treatment benefit on the primary end-point might be confounded by a placebo effect.

6.1.1 Target population and screening:

Patients with a diagnosis of IPF, or with another fibrotic ILD (including fibrotic non-specific interstitial pneumonitis, fibrotic organising pneumonia and fibrotic hypersensitivity pneumonitis) according to established guidelines, (12; 13), whose oxygen saturation (SaO_2) at rest on room air is ≥ 94% and falls ≤_88% on a baseline 6MWT will attend Visit 1 (Screening visit Week (– 2)) to undergo initial screening..

We plan to obtain informed consent and screen patients during their routine outpatient/inpatient assessment. As part of routine clinical care, all patients will undergo: past and current medical history, current concomitant medications, demographics, routine blood tests including BNP, echocardiogram, physical assessment, including oxygen saturation on room air (RA), and a six Minute Walk Test (6MWT). Historical evidence for the investigations will be acceptable as outlined in Section 10.2 of the study protocol.

Patient of childbearing potential will be tested for pregnancy during the screening visit.

6.1.2 Two weeks Run in period

Following the initial screening visit (Visit 1) subjects consented for the study will enter a two week run in period. There should be no changes to treatment or lifestyle in this period.

- Subjects who fail to meet the eligibility criteria at the end of the run-in period will be withdrawn from the study.
- Subjects who meet eligibility criteria and are deemed stable at the end of the run-in period will be assigned in random order to two weeks on ambulatory oxygen or no treatment for two weeks during the baseline visit.

6.1.3 Baseline Assessments – Visit 2 in Week 0

After the two weeks ‘run in period’ patients will return to the clinic to attend the baseline visit (study Visit 2 in Week 0).

Patient will be asked to complete the K-BILD (14) questionnaire in addition to other questionnaires (the Shortness of Breath Questionnaire (SOBQ (15)), St George’s Respiratory Questionnaire (SGRQ) (16), Hospital Anxiety and Depression Scale (HDAS)(17,18)), and to perform two 6MWTs, one on oxygen and one on air-filled canisters at the flow rate identified during the screening visit, in random order, with a rest of at least 30 minutes between tests. The patient will be blind to the content of the canisters.

Measured parameters will include 6-minute walk distance, oxygen saturation and heart rate measured continuously (WristOx2™ model 3150), Borg dyspnoea and fatigue score before and at the end of the test, time to recovery of heart rate, oxygen saturation, Borg dyspnoea and fatigue. These parameters will be related to any changes in the primary and secondary outcome variables in the current trial, to identify any baseline predictors of responsiveness.

Only patients with stable respiratory symptoms at baseline visit (no changes in medications and no chest infections during the four weeks before the baseline visit including the run in period) will be randomized into the study as outlined in Section 9.2.

6.1.4 Ambulatory Oxygen crossover Trial procedure

This study is a randomized crossover trial of ambulatory oxygen for use during physical activity. Patients meeting eligibility criteria and deemed clinically stable at the end of the run in period, will be assigned in random order to two weeks on ambulatory oxygen or not during the baseline visit. Optimal oxygen flow rates will be determined during the screening visit on the basis of the entry 6MWT on O_2, so as to maintain oxygen saturation ≥ 90 for at least half of the 6MWT, where possible. Following receipt of the treatment arm assigned during the baseline visits for two weeks patients will cross-over to receive the alternative treatment for further two weeks.

6.1.5 Ambulatory Oxygen Unit

Portable oxygen can be delivered by a variety of devices, differing in weight and size. For the purposes of this study, in order to standardize delivery and taking into account availability across oxygen company contractors, the light-weight portable oxygen gas cylinders will be used across sites to standardise mode of delivery. More information is provided in Section 13 of the study protocol.

6.2 Dosage regimen and rationale

This is a short trial as oxygen has immediate effect. The short duration was also chosen to minimise the possibility of changes to the disease status or use of other drugs, and therefore to minimize background variation in potential confounding factors. Oxygen flow rates will be determined on the basis of the entry 6MWT on O_2, so as to maintain oxygen saturation ≥90 for at least half of the 6MWT, where possible.

6.3 Concomitant treatment

It will be possible to introduce any rescue medication during the study period as judged appropriate by the treating physician. The aim is to enrol subjects whose treatment is not expected to change during the four weeks treatment period of the trial. Patients who are on established treatment for their IPF or other Fibrotic Lung Disease (FLD), with immunosuppressants, corticosteroids, NAC and Pirfenidone will continue on their regular medication. Concomitant medication will be recorded in patient’s notes and the Case Report Form (CRF).

6.4 Schematic of trial design

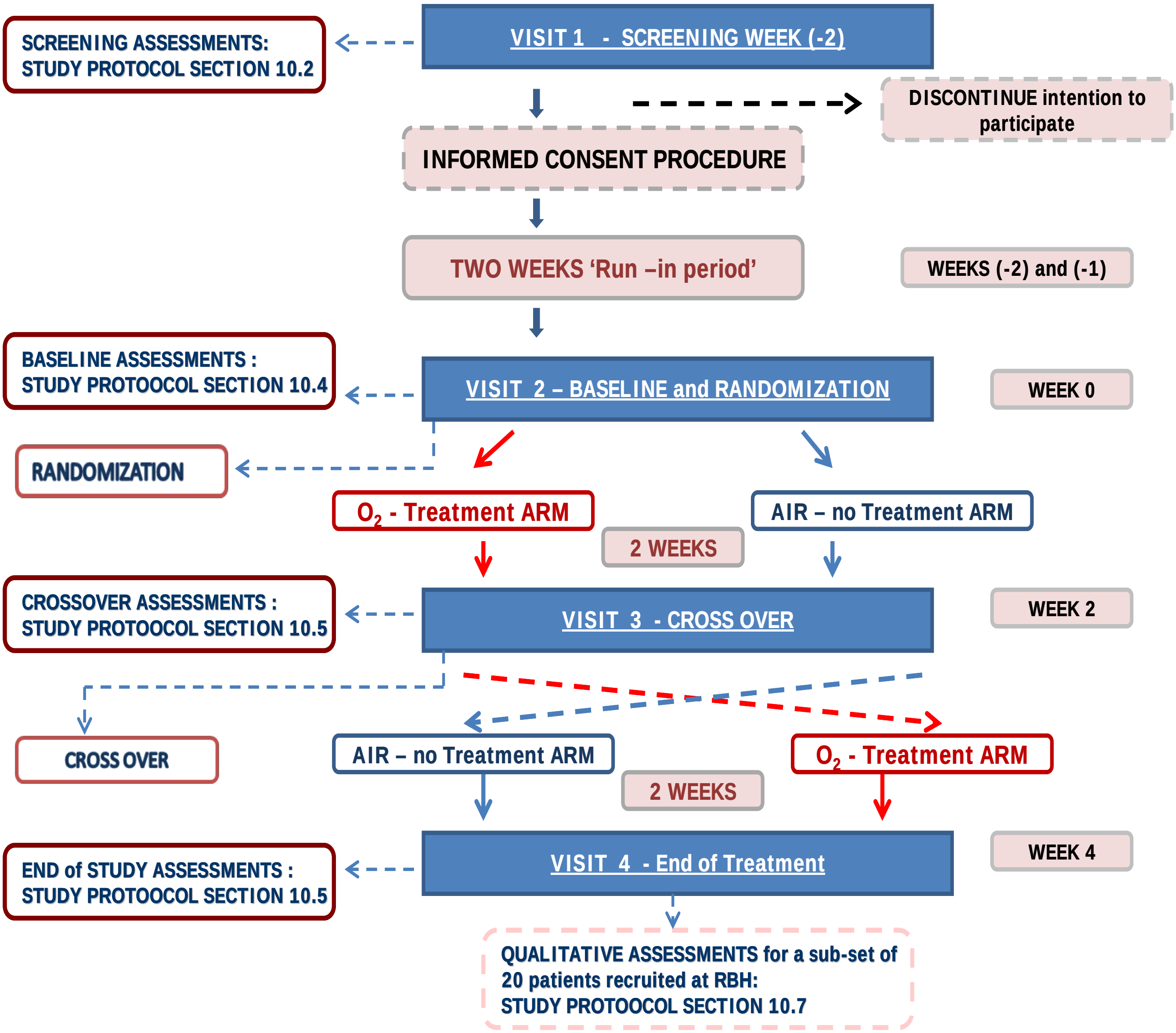

7 Eligibility criteria

7.1 Inclusion criteria

i. Patients with a diagnosis of IPF, or with another fibrotic ILD (including fibrotic non-specific interstitial pneumonitis, fibrotic organising pneumonia and fibrotic hypersensitivity pneumonitis) according to established guidelines.

ii. Patients aged 18 - 99 yrs.

iii. Patients whose oxygen saturation (SaO_2) at rest on room air is ≥94% and falls ≤ 88% on a baseline 6MWT (performed as part of their routine assessment);

iv. Patients with stable symptoms (no changes in medications and no chest infections) and treatment during the period of four weeks prior to being randomised into the study, including the two week run in period.

7.2 Exclusion criteria

i. Patients expected to change treatment over the time course of the study, and those meeting criteria for long term oxygen therapy, *i.e.* hypoxic at rest (Oxygen Saturation at rest on room air <94%).

ii. .

iii. Patients with connective tissue disease-associated ILD or with sarcoidosis with musculoskeletal/joint involvement/symptoms will be excluded due to the potential impact in relation to day-to-day mobility.

iv. Patients with significant communication or other locomotor difficulties, and/or severe co-morbidities.

v. Current smokers in view of the potential risks associated with use of supplemental oxygen.

vi. Pregnancy.

vii. History of symptomatic ischaemic cardiac disease (exertion-induced chest pain).

viii. Anaemia, Hb < 9g/dl.

8 Subject/Patient Recruitment process

Patient recruitment at a site will only commence once the trial team has ensured that the following approvals/essential documents are in place:

1. The main REC, and Clinical Trial Authorization (CTA) approval,
2. Final sponsorship and/or NHS Permission (previously known as R&D approval),
3. Sponsor has conducted the trial initiation procedure
4. Local Site Delegation of Duties and Signature Log is completed.

All sites participating in the trial will also be asked to provide a copy of the following:

1. Signed Clinical Trial Site Agreement (CTSA),
2. Host site (R&D approval) and/or NHS Permission
3. Signed Delegation of Duties and Responsibilities Log.

9 Study procedures

9.1 Informed Consent procedure

Informed consent will be obtained by the Principal Investigator (PI) and co-Investigators in the collaborating sites, and/or nominated deputies, including a Research Nurse or an ILD clinical research fellow, as recorded on Sponsor's Delegation of Responsibilities Log. Only those members of the study team who have clinical responsibility for the care of patients under the care of the general medical service will be permitted to seek informed consent. All individuals taking informed consent will have received training in Good Clinical Practice (GCP).

The potential participants will be approached in clinic by their usual care team and if they agree, they will then be approached by a member of the research team and they will have the study explained verbally and be given written information (a copy of the PIS) to take away and allowed time to consider whether to join the study. They will then be telephoned to ask if they wish to join.

Alternatively, a Patient Information Sheet (PIS) will be posted to the patient before consenting or given to them on the day of consent, at their clinical appointment. Sufficient time will be given to the patient to allow them to read the information and formulate any questions. They will then be approached at their clinic visit or by telephone to ask whether they would like to join the study.

The research clinical fellow and/or nurse and clinical team will work in conjunction to identify suitable patients. Patient records will not be accessed without the permission of the clinical team responsible for the patient.

Consent to enter this study will be obtained after a full account has been provided of its nature, purpose, risks, burdens and potential benefits, and the patient has had the opportunity to deliberate. The patient will be allowed to specify the time they wish to spend deliberating, usually up to 24 hours.

Periods shorter than 24 hours will be permitted if the patient feels that further deliberation will not lead to a change in their decision, and provided the person seeking consent is satisfied that the patient has fully retained, understood and deliberated on the information given. This provision has been made with the support of our patient advisory group.

Likewise, periods longer than 24 hours will be permitted should the patient request this. The Investigator or designee will explain that the patients are under no obligation to enter the trial and that they can withdraw at any time during the trial, without having to give a reason.

A copy of the signed Informed Consent Form (ICF) along with a copy of the most recent approved Patient Information Sheet (PIS) will be given to the study participant. The original signed consent form will be retained at the study site (one filed in the patient's medical notes and one filed in the Investigator Site File (ISF)). A copy of the consent form will also be given to the patient.

If new safety information results in significant changes to the risk–benefit assessment, the consent form will be reviewed and updated if necessary. All subjects, including those already

being treated, will be informed of the new information, given a copy of the revised consent form and asked to re-consent if they choose to continue in the study.

9.2 Randomisation procedure

All study patients will be randomized twice during the baseline study visit:

1. Randomisation as to the order in which subjects will perform the 6MWT on oxygen *versus* air. The patients will be blind to the content of the canister.
2. Randomisation as to the order in which subjects will have the portable home oxygen *versus* no oxygen during the first two weeks of treatment.

The Trust Statistician, Winston Banya, will provide a master randomization list that will form basis of the study randomization *via* an InForm database set up by Imperial College Clinical Trials Unit (ICTU).

9.3 Emergency un-blinding

Emergency un-blinding is not applicable to this study. Even though the patient will be blinded while undertaking the 6MWT on oxygen or air, the investigator will not be blind to the content of the canister.

The air or compressed oxygen used during the 6MWT are not considered to be study IMPs or NIMPs.

10 Study Assessments

10.1 Timing of Assessments

The schedule for study visits including the assessments and procedures to be performed is presented in Table 2 Schedule of events table. The scheduled study visits should occur at the specified week post-screening ± 7 days unless otherwise specified.

The primary outcome will be the change in the King's Brief Interstitial Lung Disease (K-BILD) total health status score (14). The K-BILD is a questionnaire developed and validated specifically for patients with ILD, using a cohort of 173 ILD patients attending the Royal Brompton Hospital (RBH) and King's College Hospitals (KCH) in London.

The K-BILD asks patients to rate the impact of their ILD in the previous 2 weeks. It is a simple, brief, and easy to complete questionnaire containing only 15 questions, relating to three domains: breathlessness and activities, psychological and chest symptoms. Patients are asked to rate the severity of the impact of their ILD on each of 15 items, on a scale from 1 to 7.

Repeatability (measured 2 weeks apart in patients with clinically stable ILD) is high, with an intra-class correlation coefficient of 0.94 for the total score ("health status" score), and of 0.96 for breathlessness and activity domains.

10.2 Screening assessments at Visit 1 (Week -2)

Patients with fibrotic ILD will be screened during their routine outpatient/inpatient assessment.

As part of routine clinical care, all patients will undergo:

- Physical examination (including demographics past and current medical history);
- Blood pressure;
- Pulse oxymetry at rest on room air;
- Spirometry and Echocardiogram
- Concomitant medication review;
- 6 Minute Walk Test (6MWT) - patients who desaturate ≤ 88% on room air (RA) will have a repeat 6MWT on ambulatory oxygen, to assess optimal flow (7). 6MWT measured parameters will include 6-minute walk distance, oxygen saturation and heart rate Borg dyspnoea and Borg fatigue score before and at the end of the test, time to recovery of heart rate, oxygen saturation, Borg dyspnoea and Borg fatigue score, acceptable also +/- 1 week.;
- Collection of blood for laboratory analysis including BNP level. BNP levels are acceptable within +/- 1 month from visit 1;
- Pregnancy test (for women of child bearing potential).

Historical evidence will be acceptable for the purpose of study screening only if it was conducted within the timelines given below:

- The echocardiogram and the spirometry will be accepted if performed within 6 months before screening visit (visit 1). Where either of the assessments are not available as part of routine medical care the assessment should be booked to take place within 6 weeks post visit 1.
- The 6MWT will have to be performed at the screening visit +/- one week.
- BNP levels are acceptable within +/- 1 month from the visit 1.

Patients who desaturate ≤ 88% will have a repeat 6MWT on ambulatory oxygen, to identify the oxygen flow needed to maintain optimal saturation (≥ 90%, for at least half of the 6MWT duration, where possible) (7).

Patient should rest for at least 20 minutes between walks. The initial amount of oxygen offered will be calculated depending on level of desaturation during 6MWT on room air, as follows:

- Saturations 88-86% - start with 2 litres,
- Saturations 86-83% -start with 3 litres,
- Saturations 83-80% - start with 4 litres,
- Saturations 80-78% - start with 5 litres,

For saturations < 78%, start with 6 litres and titrate up to and including 10 litres as required to achieve saturations of 90%, for at least half of the 6MWT duration, where possible.

Every 2 minutes of the 6MWT, the operator should consider increasing the O_2 flow, depending on saturation. If the saturation reached is between 87-89%, the flow should be increased by 1L/min, if < 87% by increments of 2L/min.

If SaO_2 is still < 88% for most of the walking test on 6L/min, a further 6MWT starting from 8L/min should be performed, using up to a maximum of 10L/min.

Oxygen saturation should be monitored for at least 3 minutes post walk and/or until saturation value has come back to pre-test value.

Patients expected to change treatment over the time course of the study, and those meeting criteria for long term oxygen therapy with SaO_2 at rest on room air < 94% will be excluded.

Patients with connective tissue disease-associated ILD or with sarcoidosis will be excluded if they have musculoskeletal involvement because of the potential impact of extrapulmonary involvement in day-to-day mobility, as will patients with significant communication or other locomotor difficulties, and/or severe co-morbidities, history of symptomatic ischaemic cardiac disease (exertion-induced chest pain) and anaemia (Hb < 9g/dl).

Patients included in the study will be given the K-BILD questionnaire during the screening visit to complete within 48h of the screening visit and to bring it back when attending Visit 2.

Patients included in the study will be given K-BILD questionnaire during the screening visit to complete within 48h of the visit and to bring it back at Visit 2 (Week 0) following the run in period.

10.3 Two weeks 'run in period'

Subjects will enter a two week run in period and those who fail to meet the eligibility criteria at the end of the run-in period will be withdrawn from the study.

10.4 Baseline assessments and Randomization (Visit 2 in Week 0)

Following the run-in period at the baseline visit (visit 2), stability of respiratory symptoms will be assessed according to routine clinical care. Only patients with stable symptoms (no chest infections and no changes in medications) during four weeks before including the run in period will be assigned in random order to two weeks on ambulatory oxygen or not treatment for two weeks.

Patients will be invited to perform two 6MWTs, one on oxygen and one on air-filled canisters at the flow rate identified during the first session, in random order, with a rest of at least 20 minutes between tests. Oxygen flow rates to be provided to the patient will be determined during the screening visit on the basis of the 6MWT on O_2, as the flow needed to provide and to maintain optimal oxygen saturation ≥ 90% for at least half of the 6MWT duration, where possible, and/or a maximal flow of 10L/min (7).

The patient will be blind to the content of the canisters.

Patients will then be randomised as to the order in which they will receive the portable oxygen *versus* no oxygen for the first two weeks of the treatment period as outlined in Section 9.2.

The treatment period of the study will consist of a four week randomized crossover trial of ambulatory oxygen for use during physical activity. At Visit 2, eligible patients will be asked to:

- complete the K-BILD questionnaire(14) (see “primary outcome” section) and three other questionnaires (the Shortness of Breath Questionnaire (SOBQ) (15), St George’s Respiratory Questionnaire (SGRQ) (16), Hospital Anxiety and Depression Scale (HDAS) (18), see below section 10.5);
- Concomitant medication review;
- Adverse Event
- Pulse oximetry at rest on room air
- 6MWT one on oxygen at the optimal flow rate identified during the screening visit
- 6MWT on air

The two 6MWTs (one on oxygen the other on air cylinder) will be undertaken in random order, with a rest of at least 30 minutes between tests. The patient will be blind to the content of the canisters. 6MWT measured parameters will include 6-minute walk distance, oxygen saturation and heart rate measured continuously (WristOx2™ model 3150), Borg dyspnoea and Borg fatigue score before and at the end of the test, time to recovery of heart rate, oxygen saturation, Borg dyspnoea and Borg fatigue.

- Provide a Sensewear armband for patients to wear during the second week (5+2 days) of each of the two week treatment periods;
- Provide oxymeter for patients to wear for 48 hrs during the second week of each two week treatment periods (on oxygen and on no oxygen);
- Supply a diary of daily activities and instructions on completion by patients during the two weeks until the next visit (Visit 3);
- Dispense treatment based on randomisation allocation.
- The next study visit will be set up within two weeks of the baseline visit.

10.5 Crossover Visit – Visit 3 in Week 2

At Visit 3, patients will be asked to:

- Pulse oximetry at rest on room air;
- Concomitant medication review;
- AE assessment;
- Global assessment of change in walking ability and breathlessness (18);

- Ask patients to submit daily activity diary relating to the previous two weeks, Sensewear armband given during the baseline visit (visit 2) and therefore related to the past two weeks;
- Ask patients to submit their 48 hrs oxymeter readings, provided at the baseline visit;
- Complete study questionnaires including K-BILD questionnaire (14), Shortness of Breath Questionnaire (SOBQ) (15), St George's Respiratory Questionnaire (SGRQ) (16), Hospital Anxiety and Depression Scale (HDAS) (18),
- Ask patients to complete diary of daily activities during the following two week treatment period until the next visit (visit 4);
- Provide Sensewear armband for patients to wear during the second week (5+2) of the two weeks of the final treatment period;
- Provide oxymeter for patients to wear for 48 hrs during the week when the Sensewear armband is worn;
- Cross-over to the alternative treatment.

10.6 End of Treatment Visit – Visit 4 in Week 4

At Visit 4, patients will be asked to undertake:

- Pulse oximetry at rest on room air
- Concomitant medication review
- AE assessment
- Global assessment of change in walking ability and breathlessness (19)
- Ask patient to submit daily activity diary, Sensewear armband, and 48 hrs oxymeter reading related to the final two week treatment;
- Complete study questionnaires including K-BILD questionnaire (14), Shortness of Breath Questionnaire (SOBQ) (15), St George's Respiratory Questionnaire (SGRQ) (16), Hospital Anxiety and Depression Scale (HDAS) (18).

10.7 Qualitative assessments

Qualitative assessment of impact of ambulatory oxygen will be undertaken *via* a semi-structured interview, with a purposive sample of 20 patients to investigate patients' and their carers' personal perspective on how the ambulatory oxygen has affected their day to day life. The interview will be conducted within 2 weeks of the end of treatment visit. Patients from the RBH site will be approached by the researcher at the end of treatment visit and asked if they will participate in the qualitative assessment. The PIS given to all patients at the beginning of the study is designed to explain the qualitative assessment aspect of the study. All patients recruited at RBH will be informed that the researcher will approach them during the last patient visit to ask if they wish to be interviewed to include their views of the impact of the ambulatory oxygen in the qualitative aspect of the study. If

the patient agrees a date will be agreed for the patient to be interviewed at a venue convenient to them (usually the patient's home).

During the interviews a semi-structured topic guide will address practical barriers to optimal oxygen usage, practical, social and psychological difficulties encountered, concerned about dependency, and views on the information required prior to ambulatory oxygen prescription. The interviews will also explore patients' (and carers') experience of participating in the trial. Notes will be written after each interview to aid reflexive analytical processes (20). Individual feedback on how the system and the service could be improved will assess how the needs of patients and their future involvement can be incorporated into the design of more patients and their future involvement can be incorporated into the design of more patients' centred devices.

Qualitative interview analysis: Interviews will be transcribed verbatim. Interview transcripts will be analysed thematically using a framework approach (21). Atlas/ti computer software (http://atlasti.com) will be used to manage and index the data prior to charting, mapping and interpretation.

10.8 Unscheduled Visits

Patients may require an unscheduled visit in addition to the regular scheduled protocol visits (*e.g.* symptoms of infection, worsening of disease, or assessment of AEs). If a patient requires an unscheduled visit, the study centre will be strongly encouraged to undertake the following assessments:

- Physical examination
- Vital signs
- AE review
- Concomitant medication review
- Lung function (spirometry and blood gas analysis)
- Collection of blood for laboratory analyses (to include full blood count)

10.9 Summary of study Assessments

Questionnaires: As previously stated, the primary outcome will be the change in the King's Brief Interstitial Lung Disease (K-BILD) total health status score (14). Participants will be asked to complete this questionnaire, at screening and baseline visit (Visit 1 and Visit 2) and at the end of each two-week period (visit 3 and 4). In the same points (visit 2, 3 and 4) they will also be asked to complete the Shortness of Breath Questionnaire (SOBQ - University of California, San Diego) (15), a widely validated index, designed to assess activity-related dyspnoea, as well as a questionnaire evaluating Quality of Life (St George Respiratory Questionnaire – SGRQ) (16) and the Hospital Anxiety and Depression scale, both validated in ILD (18).

At the end of the third and fourth visit, participants will also be asked to make a global assessment of change in their walking ability and in their breathlessness on walking as “worse”, “about the same” or “better” (19), when compared to the previous two weeks.

Activity assessment: During the second week of each of the two week periods of the study treatment, patients will be asked to wear an activity monitor during waking hours, the Sensewear armband (Bodymedia - Pittsburgh, Pennsylvania), which measures energy expenditure, daily number of steps, and time spent at different levels of physical activity. Sensewear-derived measurements are sensitive and repeatable in chronic lung disease patients (22). The monitor is currently used at the RBH in a number of projects in COPD, and Dr Hopkinson, one of this proposal's co-applicants, has established expertise in the use and assessment of activity measures. Patients will also be asked to complete a diary of daily activities as measure of physical activity, by using the modified diary method of Follick *et al* () (23, 24). Participants will be asked to complete this diary at least 3 times daily, recording the activity undertaken for the majority of each two hour block, in addition to specifying whether oxygen cylinders were used. In addition, total hours of outings each day will be recorded.

Oxygen Cylinder Use: will be expressed as number of cylinders used and as hours of use from self-reported data; patients will also be asked to write down the time of use of oxygen canisters for the two days a week when they will also be wearing the portable oxymeter, so as to correlate the two. At the end of the two weeks on oxygen, each patient will also be asked to fill in questionnaires about the number of cylinders fully and partially used and the number of unused cylinders.

Oxygen saturation: two days a week for each of the four weeks, patients will also be asked to wear a portable oxymeter, to assess oxygen saturation for 48 continuous hours. Measures of continuous oxygen saturation will also allow control for self-reported use of ambulatory oxygen.

10.10 Summary flow chart of study assessments

A summary chart of study assessments can be found in Appendix 1 of the study protocol.

11 Methods

11.1 Laboratory procedures

Blood samples will be taken at the start of the four week period including serum BNP levels. The measurement will be performed by the routine hospital laboratory within the trial sites as a standard clinical procedure in line with local policies and procedures. Copies of local reference ranges will be collected and the investigator must notify the CI of any subsequent changes in individual patient normalised values. These changes should also be recorded in the eCRF.

11.2 Radiology or any other procedure(s)

No additional x-rays, CT scans or exposure to radiation will occur as a result of taking part in this study.

11.3 Definition of the End of Trial

The end of the trial will coincide with the Last Patient Last Visit (LPLV).

12 Discontinuation/withdrawal of participants and stopping rules

As we do not expect there to be any toxicity from the use of ambulatory oxygen, subjects will be withdrawn from the trial only if they wish to do so. We will ask any patient who wishes to withdraw from the study, if they are willing, to fill in the relevant questionnaires at the end of the two and four week period, if needed *via* telephone, so as to be able to compare change in health status and other questionnaire related outcomes compared to baseline, against subjects completing the trial.

As our power calculations have allowed for subject withdrawals and incomplete data in a subset of patients, we do not expect the need to replace subjects, unless < 50 complete the study.

We do not envisage prematurely stopping the trial, again in view of the lack of toxicity of ambulatory oxygen in the patients selected for this trial.

13 IMPs and non-IMPs used in the trial

13.1 Name and description of each IMP

The IMP investigated in this study is medical oxygen (PL 15929/005), manufactured by Air Liquide Ltd. UK. Oxygen is a colourless, odourless gas with molecular weight 32, a boiling point of 183.1°C (at 1 bar) and a density of 1.355 kg/m^3 (at 15°C and 1013mb).

Oxygen is present in the atmosphere at 21% and is an absolute necessity for life. Each of the four oxygen companies across the UK provide a light and a standard weight oxygen cylinder.

For the purpose of this study, we plan to use light-weight cylinders, which vary in weight from 1.8 kg (provided by BOC) to 2.2 Kg (provided by Dolby Vivisol and Air Liquide). We have decided to opt for the oxygen gas cylinders rather than the liquid oxygen counterparts, as the use of the former will allow an independent check on the number of oxygen cylinders used per week. This would not be possible using liquid oxygen, as the liquid oxygen contained in the cylinders evaporates over time even if not used by the patient, and there is no way for the investigators of independently counting the number of cylinders used, as the patient would fill in the liquid oxygen from a storage unit. Although there is the potential issue of difference in weight between the cylinders provided by Airliquide (which we expect would cover approximately 60% of our cohort, from a review of our referral patterns per UK region) and the other oxygen companies, we plan to adjust for cylinder weight in the analysis. Further, the relative weight of an oxygen cylinder will in any case vary widely depending on the height and weight of the patient and we plan to adjust for these variables in the analysis. Light weight cylinders across the different oxygen companies provide oxygen

flow rates from 0.5 to 15 L/min, lasting from 10 to 2 hours at a time depending on oxygen flow. Continuous oxygen flow *via* nasal cannulae will be used for all patients to standardize mode of delivery.

Ambulatory oxygen will be supplied by the relevant oxygen companies, on a Home Oxygen Order Form (HOOF); the amount of oxygen required (hours per day) and flow rate should be specified. The supplier will determine the appropriate equipment to be provided. Special needs or preference will be specified on the HOOF. The clinician will obtain patient's consent to pass on the patient's details to the supplier and the fire brigade. The supplier will contact the patient to make arrangements for delivery, installation, and maintenance of the equipment. The supplier will also train the patient to use the equipment. The supplier will continue to provide the service until the revised order is received or until notified that the patient no longer requires the home oxygen service.

Overall there are four oxygen companies which provide supplemental oxygen in the UK, including Air Liquide (which services North and South London, North and South West England, and the East Midlands), BOC (Eastern England and NorthEast), Baywater (Yorkshire and Humberside, West Midlands and Wales) and Dolby Vivisol (South Central England and South East Coast).

Source of IMPs including placebo

Ambulatory oxygen will be provided by the relevant oxygen companies in the UK based on the location of the patient participating in the study. It is currently confirmed that all three sites are in the region of the UK supplied by Air Liquide. However, the relevant oxygen company, dependent on the patient's post code, will be used as part of the standard clinical home delivery of ambulatory oxygen (*i.e.* BOC, Dolby Vivisol, and Baywater).

Ambulatory oxygen is also available on the drug tariff when a patient's home and social set up are appropriate. The most common portable flask is the Helios Marathon (H850) which can provide 1 - 6Lpm constant flow, or 1.5 - 4 on a pulsed delivery. The patients can refill the flask themselves. Liquid oxygen generally is lighter than portable gas cylinders.

Patients will also be supplied with a nasal cannula for oxygen delivery.

13.2 Accountability procedure for the IMPs

Ambulatory oxygen will be provided by the existing structure using the oxygen companies as described in Section 14.1 above. At the end of the study period, patients will continue on ambulatory oxygen as part of their standard care, and there will therefore be no returned IMP. If the patients are randomised to start on oxygen on the first two weeks of the four week trial period, the company will be asked to remove any unused oxygen cylinders prior the two weeks "off" oxygen.

To determine oxygen use during the study and in particular during the 2 weeks when patients are randomized to be on treatment, patients will be asked to keep a daily diary recording the number of hours spent using oxygen. The flow will be fixed at the optimal flow established at the baseline 6MWT on O_2 at the study entry. Investigators will also ask the relevant oxygen company to provide the number of cylinders delivered to the patient per

week along with the number of any unused cylinders at the end of the two weeks on oxygen, where possible.

The pharmacy hospital department will not be responsible for the provision and management of study IMP.

13.3 Route of administration, dosage, dosage regimen, and treatment period(s) of the IMPs

Ambulatory oxygen as provided by light weight portable oxygen cylinders, will be set up to provide an optimal oxygen flow rates established on study entry. The flow rate to be used for the individual patient will be the one identified in the baseline 6MWT on ambulatory oxygen, as the flow necessary to maintain optimal saturation during the walk test. We do not envisage that the flow rate will change during the two weeks of the trial.

13.4 Dosage modifications

As explained in more detail previously, the study will consist of two weeks on the ambulatory oxygen compared to two weeks without the portable unit. Should the patient require the change in flow rate this will be recorded in patients' notes and the CRF.

13.5 Assessment of compliance

Oxygen use will be expressed as number of full, half full and unused cylinders as self-reported by the patient after the two weeks on oxygen, which will be crossed checked by assessing accountability record provided by the relevant oxygen company.

To validate patient-reported use of oxygen cylinders, a research nurse at the Aintree site will sample 10 consecutive patients living in the nearby area. The research nurse will visit patients at home at the end of the two weeks of ambulatory oxygen to independently access numbers of fully used, half used and full oxygen cylinders. Comparison between patient and nurse reported cylinders use will be included in data analysis.

Patients will also be asked to fill in a daily oxygen use diary card to write down the time of use of oxygen canisters, and the activity being performed. This will be crossed check with the continuous oxygen saturation data recorded by the portable oxymeter, which they will be asked to wear for two days a week, so as to correlate the two.

13.6 Post-trial IMP arrangements

At the end of the trial, all patients will be offered to continue on ambulatory oxygen, as they would have been if they had not taken part in the trial.

13.7 Name and description of each non-IMP (NIMP)

Not applicable.

14 Pharmacovigilance

14.1 Definitions

Adverse Event (AE) — any untoward medical occurrence in a patient or clinical trial subject who is administered an IMP and which does not necessarily have a causal relationship with this treatment. (*i.e.* any unfavourable or unintended change in the structure (signs), function (symptoms), or chemistry (lab data) in a subject to whom an IMP has been administered, including occurrences unrelated to that product)

Adverse Reaction (AR) — any untoward and unintended responses to an IMP related to any dose administered (*i.e.* any unfavourable or unintended change in the structure (signs), function (symptoms), or chemistry (lab data) in a subject to whom an IMP has been administered and related to any dose administered)

Serious Adverse Event (SAE) or Serious Adverse Reaction (SAR)—any Adverse Event or Reaction in a trial subject that:

- Results in death; or
- Is life-threatening (places the subject, in the view of the Investigator, at immediate risk of death)
- Requires hospitalisation or prolongation of existing hospitalisation (hospitalisation is defined as an inpatient admission, regardless of length of stay; even if it is a precautionary measure for observation; including hospitalisation for an elective procedure, for a pre-existing condition)
- Results in persistent or significant disability or incapacity (substantial disruption of one's ability to conduct normal life functions)
- Consists of a congenital anomaly or birth defect (in offspring of subjects or their parents taking the IMP regardless of time of diagnosis).

Important medical events that may not be immediately life-threatening or result in death or hospitalisation but may jeopardise the subject or may require intervention to prevent one of the outcomes listed in the definition of serious will also be considered serious.

Suspected Unexpected Serious Adverse Reaction (SUSAR) — an Adverse Reaction which is classed in nature as both serious and unexpected.

An Unexpected Adverse Reaction is an Adverse Reaction, when both the nature and severity of the event is not consistent with the information about the medicinal product in question, as set out below:

(a) in the case of a product with a marketing authorization, in the Summary of Product Characteristics (SmPC) for that product,
(b) in the case of any other IMP, in the Investigator's Brochure relating to the trial in question.

14.2 Terminology for classification of SAEs

A simple and brief description of **clinical symptoms** should be given using the following descriptions to record information about AEs:

<u>1. Severity</u> will be described using the following categories:

- **Mild**—the adverse event does not interfere with the volunteer's daily routine, and does not require intervention; it causes slight discomfort.
- **Moderate**—the adverse event interferes with some aspects of the volunteer's routine, or requires intervention, but is not damaging to health; it causes moderate discomfort.
- **Severe**—the adverse event results in alteration, discomfort or disability which is clearly damaging to health.

2. Relationship to treatment — the assessment of relationship of AEs to the administration of IMP is a clinical decision based on all available information at the time of the completion of the CRF. The following categories will be used:

- **Definitely** — there is clear evidence to suggest a causal relationship, and other possible contributing factors can be ruled out.
- **Probably** — there is evidence to suggest a causal relationship, and the influence of other factors is unlikely.
- **Possibly** — there is some evidence to suggest a causal relationship (e.g. the event occurred within a reasonable time after administration of the trial medication). However, the influence of other factors may have contributed to the event (i.e. the patient's clinical condition, other concomitant events).
- **Unlikely** — there is little evidence to suggest there is a causal relationship (e.g. the event did not occur within a reasonable time after administration of the trial medication). There is another reasonable explanation for the event (*e.g.* the patient's clinical condition, other concomitant treatments).
- **Not related** — there is no evidence of any causal relationship.
- **Not Assessable**

3. Expectedness will be described using following categories:

- **Expected** — an AE that is classed in nature as serious and which is consistent with the information about the IMP listed in the Investigator Brochure (or SmPC) **or clearly defined in this protocol.**
- **Unexpected** — an AE that is classed in nature as serious and which is not consistent with the information about the IMP listed in the Investigator Brochure (or SmPC).

14.3 Recording of Safety Information

Collection, recording and reporting of AEs (including serious and non-serious events and reactions) to the Sponsor will be done according to the Sponsor's Pharmacovigilence SOP.

14.3.1 Adverse Events (AEs)

All Adverse Events will be recorded in the hospital notes in the first instance.

A record of all AEs, whether related or unrelated to the treatment will also be kept in the CRF.

If the Investigator suspects that the disease has progressed faster due to the administration of the IMP, then he/she will report this as an unexpected adverse event to the sponsor.

14.3.2 Serious Adverse Events (SAEs)

All SAEs will be recorded in the hospital notes and the eCRF.

All SAEs will be reported to the Sponsor *via* the Inform System on an SAE form unless otherwise stated in the protocol. The Chief or Principal Investigator will complete the Inform SAE reporting form and the form will be faxed to the RO on 020 351 8578 or E-mailed to safetyreporting@rbht.nhs.uk, *via* Inform system within 24hrs of the Investigator becoming aware of the event.

The Chief or Principal Investigator (CI/PI) will respond to any SAE queries raised by the Sponsor as soon as possible.

Medical oxygen is licensed in the UK and will be used within its marketing authorization in this study. The most up to date version of the SmPC will be used as the IMP reference document for this study.

14.3.3 Specific Adverse Events to be captured

At each visit patients will undertake study related assessment to check for adverse events. The data manager will perform regular checks which will be reviewed by the CI and DMC reports, on the eCRF and produce reports were the following parameters have exceeded an acceptable value.

Specific AE's that require recording on the eCRF include:

- Chest infections
- Headache(s)
- Disease Progression Expected SAE/Rs

14.3.4 Expected SAE/Rs related to IMP

Expected Adverse Reactions are known side effects of the IMP reported in SmPCs of Medicinal Oxygen.

Ambulatory oxygen therapy is usually associated with only mild side effects, which may include nasal mucosa dryness or skin irritation from the nasal cannula.

14.3.5 Expected SAEs related to underlying disease

Events that are expected and related to underlying disease include study endpoints, disease progression, chest infections and death secondary to disease progression.

14.4 Reporting Adverse Events (AEs) – (including SAE/Rs)

a) There is routine monitoring of patient status, blood tests and lung function and data will be captured on the eCRF.

b) All SAEs/SARs (including expected) will be recorded on the eCRF by the investigator/or designee within 24 hours of their becoming aware of the event. The eCRF will send a notification of each SAE report to the Research Office (*via* email detailed in section 15.3.2) and the CI (or designee) who will review the report within 2 working days of receipt.

c) Prolongation of hospital admission or new hospital admission for infections which are related to IMP and or the underlying disease progression investigators are required to report within 7 days of their becoming aware of the event.

d) All SAEs, whether related or unrelated to the treatment will be recorded in the hospital notes and eCRF. The Sponsor will have access to SAEs *via* the eCRF.

e) If the Investigator suspects that the disease has progressed faster due to the administration of the IMP, then s/he will report this as an unexpected AE.

14.4.1 SUSARs

All SUSARs will be notified to the Sponsor immediately or at least within 24hrs of the Investigator becoming aware of the event. Investigators must complete the appropriate SAE form on the eCRF and an automatic email notification will be sent to the Research Office and the CI immediately. The Research Office on behalf of the Sponsor will ensure that the report is reviewed by the CI (or designee) within 2 days to ensure it constitutes a SUSAR and requires expedited reporting by the Research Office.

All SUSAR will be disseminated to study PIs and participating sites *via* the research team at RBH.

14.5 Notification of deaths

We do not expect any deaths to occur as a consequence of ambulatory oxygen. However, all deaths, including deaths unrelated to the IMP, even if they occur earlier than expected, will be reported to the CI and the study Sponsor within 24 hours of the PI becoming aware.

14.6 Reporting to Regulatory Authority

The RO will notify all SUSARs to the MHRA electronically *via* e-SUSARs Database and the main REC using the CIOMS form. The CIOMS form should be completed by the CI and PI at each site and sent to the RO as described in Section 15.3.2 of the protocol.

The RO will inform the MHRA and the main REC of fatal or life threatening SUSARs as soon as possible, but no later than 7 calendar days after the RO receives the SAE report form. Any additional information will be reported within 8 days of sending the first report.

The RO must report all other SUSARs and safety issues to the MHRA and main REC, as soon as possible but no later than 15 calendar days after the RO has first knowledge of the minimum criteria for expediting reporting.

14.7 The type and duration of the follow-up of subjects after AEs

Any SUSAR related to the IMP will need to be reported to the Sponsor irrespective of how long after IMP administration the reaction has occurred.

14.8 Development Safety Update Reports

The CI or a delegated PI will prepare the DSUR, using the Sponsor's DSUR template and in accordance with the Sponsor's DSUR SOP. It will be reviewed by the Sponsor and when necessary be referred to an independent committee (*i.e.* Clinical Trials Oversight Committee (CTOC)). The RO will provide the main REC and the MHRA with a copy of the study DSUR.

14.9 Annual Progress Reports (APRs)

The Chief Investigator will prepare the APR. It will be reviewed by the JRO and sent to the main REC within 30 days of the anniversary date on which the favourable opinion was given by the Ethics committee, and annually until the trial is declared ended.

14.10 Pregnancy

There are no known side effects and/or harmful effects of ambulatory oxygen during pregnancy. In the unlikely event of a pregnancy occurring during the 4 week trial period while patients are receiving treatment as part of the study (IPF and other idiopathic fibrotic lung diseases are more common in older age groups and in males), the pregnant patient will not be removed from the trial. If the discovery of the pregnancy were to fall within the two weeks of no ambulatory oxygen the patient would be advised to continue with the prescribed ambulatory oxygen, to avoid hypoxia to the foetus.

As oxygen is not a drug, follow up of the mother and child would follow standard of care, and there would be no need for follow up specific to the ambulatory oxygen. However, as part of routine clinical care of a pregnant patient with significant lung fibrosis and gas exchange abnormalities, the patient would be referred to a centre with expertise in management of pregnancies in the context of significant lung disease, and she would remain under close follow up of the Interstitial Lung Disease Unit (ILDU) in the relevant centre, in the period up to and following delivery of the baby.

Despite ambulatory oxygen being beneficial during pregnancy, all pregnancies will be reported to the study Sponsor as described in Sponsor's PVG SOP. Patients will be excluded from the study but will be invited to attend future study visits. The specialist ILDU will be asked to provide follow up information to the CI regularly and on request until the delivery of the baby.

14.11 Reporting Urgent Safety Measures

Regulation 30 of the Medicines for Human Use (Clinical Trials) Regulations 2004 [Statutory Instrument 2004/1031], as amended by Statutory Instrument 2006/1928 states “the Sponsor and the Investigator may take appropriate urgent safety measures in order to protect the subjects of a clinical trial against any immediate hazard to their health or safety. If measures are taken, the Sponsor shall immediately and in any event no later than 3 days from the date the measures are taken, give written notice to the MHRA and the relevant REC of the measures taken and the circumstances giving rise to those measures.”

In order to prevent any delays in the reporting timelines the Sponsor has delegated this responsibility to the CI/PI. Therefore the CI/PI must report any urgent safety measures to the MHRA directly, and in parallel to the Sponsor.

14.12 Notification of Serious Breaches of GCP and/or the protocol

Any Protocol Deviations, Violations, Potential Serious Breaches and Urgent Safety Measures will be recorded using the Sponsor’s Log issued during the Sponsor’s Trial/Site Initiation meeting/visit.

Regulation 29A of the Medicines for Human Use (Clinical Trials) Regulations2004 [Statutory Instrument 2004/1031], as amended by Statutory Instrument2006/1928, contains a requirement for the notification of “serious breaches” of GCP or the trial protocol:

(1) The Sponsor of a clinical trial shall notify the licensing authority in writing of any serious breach of:

(a) The conditions and principles of GCP in connection with that trial; or

(b) the protocol relating to that trial, as amended from time to time in accordance with regulations 22 to 25, within 7 days of becoming aware of that breach.

(2) For the purposes of this regulation, a “serious breach” is a breach which is likely to effect to a significant degree:

(a) The safety or physical or mental integrity of the subjects of the trial; or

(b) The scientific value of the trial.

The Sponsor will be notified immediately of any case where the above definition applies during the trial conduct phase. The Sponsor’s SOP on the Protocol Violation/Deviations and Serious Breaches will be followed.

15 Data management and quality assurance

15.1 Confidentiality

All data will be handled in accordance with the Data Protection Act 1998.

The Case Report Forms (CRFs) will not bear the subject’s name or other personal identifiable data. The subject’s initials, and trial Identification Number (ID), will be used for identification.

15.2 Source Documents

The source documents for the trial data will be identified before the study start by the CI. It is anticipated that the majority of source data (medical progress notes and letters, tests and investigations) will be filed in the individual patients medical records. Any deviation from source data being present in the medical notes will be identified and documented for the duration of the study. The eCRF and source documents must be available at all times for review by the Sponsor's clinical trial monitor, auditors and for inspection by the Medicines Health Regulatory Agency (MHRA). The accuracy of eCRF data will be verified by review of the source documents and details will be provided in the study monitoring report for each study site.

Information should be entered legibly. If there is an error in the notes, it should be crosses through with a single line in such a way to ensure that the original entry can still be read. The correct entry should be clearly inserted. The amendment should be initialled and dated by the person making the correction immediately. Overwriting of use of correction fluid will not be permitted.

15.3 Data collection tool

Case Report Forms (CRFs) will be designed by the CI in liaison with Imperial College clinical Trials Unit (ICTU) and the final version will be approved by the Sponsor. All data will be collected on an electronic CRF (eCRF) *via* InForm database system. The eCRF will be designed in accordance with the requirements of the clinical trial protocol and will comply with regulatory requirements. Local personnel will be trained on the Inform system. Access will be restricted to site personnel, trial managers, trial monitors, data management team. Personnel will have individual logon and passwords. It is the Investigator's responsibility to ensure the accuracy of all data entered and recorded in the eCRFs. The Delegation of Responsibilities Log will identify all trial personnel responsible for data collection, entry, handling and managing the database and the data entered in the eCRF. Trial monitors will check the accuracy of the eCRF data against source documents.

The majority of source data (medical letters and progress notes, results of tests and investigations) will be filed in the individual patients' medical records. The data specifically relative to the study including questionnaire data, patient activity diaries, and readings from activity monitors/oxymeters will be entered directly onto the CRF.

All the questionnaires used in this study have been previously standardized and validated (14-19). Similarly, data obtained from the oxymeter reading and Sensewear armband have been validated as described in the literature (22-23). The patient activity diary has previously been validated against activity monitors in patients with chronic lung disease (23,24).

In order to maximize completeness of data, patients will be called at the end of each week. If they have forgotten to wear the activity monitor and/or the oxymeter for the allocated time, they will be asked to make up for the lost days over the following week. Patients will also be encouraged to continue filling in their daily activity diaries. The accuracy of the CRF data will be verified by review of the source documents.

15.4 Data handling and analysis

The InForm system (database) provided by the Imperial Clinical Trials Unit (ICTU) will be used for data entry and storage. The ICTU will design and build the electronic Case Report Forms (eCRFs) and validation rules for data entry to ensure the data can be collected accurately and stored securely. User support is provided during the life cycle of the trial to deal with forgotten passwords, questions on how the system works and any perceived issues with the system. ICTU database team also provides assistance with ad hoc report development and research data extracts for safety committees, final reports or as requested.

The service is provided using InForm Integrated Trial Management (ITM) System, a web-based data entry system which builds an Oracle database for each individual clinical trial. InForm, supplied by Oracle | Phase Forward, is widely used across the pharmaceutical industry. The databases are hosted on Imperial College's infrastructure and can be analyzed using an inbuilt reporting package, Cognos.

Central Designer, also supplied by Oracle | Phase Forward, is the tool used to design and build the eCRFs (electronic case report forms) and data entry rules for trials.

The quality of data entry will be checked at regular intervals throughout the trial. The analysis will be performed by the CI and the PIs in collaboration with the Trust statistician and will be performed independently of data entry.

16 Archiving arrangements

The trial documents (including the Trial Master File (TMF), Case Report Forms (CRFs), Informed Consent Forms along with the trial database) will be kept for a minimum of five years. They will be stored in locked offices within the Royal Brompton and Harefield NHS Foundation Trust site for the duration of the study. The Chief Investigator is responsible for the secure archiving of trial document. The trial database will also be kept electronically on the Trust computer network, for a minimum of five years.

The approved repository for longer retention of local materials for studies that involve RB&HFT patients is **Box-It Storage UK**. The study documentation will be prepared for archiving by the research team in line with the Research Office Archiving SOP and the transfer will be arranged by the Research Office.

17 Statistical design

17.1 Statistical input in trial design

Data analysis will be performed by the Chief Investigator (CI) with the input of the Trust statistician, Winston Banya, who will ultimately be responsible for the accurateness of data analysis. All data will be analysed with intention to treat and patients who do not adhere to the study protocol correctly will be excluded as “per protocol” analysis.

17.2 Endpoints

17.2.1 Primary endpoints

The primary endpoint will be the change in health status, as measured by the K-BILD questionnaire (14). It is a continuous variable.

17.2.2 Secondary endpoints

a) Dyspnoea scores as assessed by the San Diego shortness of breath questionnaire (15)

b) Global assessment of change in walking ability and exertional breathlessness as better, the same or worse

c) Quality of life as assessed by SGRQ (16) and the hospital anxiety and depression score (18)

d) Sense wear activity monitor measures of daily number of steps walked, daily expenditure and time spent at different levels of activity

e) Continuous oxygen saturation for 48 hours at weekly intervals

f) Individual questions specifically related to activity from both the K-BILD and the SOBQ questionnaires

All of the above are continuous variable and will be analysed as such at least in the first instance. However, categorical analysis will also be performed, when appropriate on reviewing the distribution of the data.

Qualitative assessment of the impact of ambulatory oxygen. With regards to the qualitative arm of this study, we plan to investigate patients' and their carers' personal perspectives on how the ambulatory oxygen has affected their day to day life by conducting semi-structured open-ended interviews in a subset of 20 patients and their carers at the end of the study. Based on the information we gained from informal conversations with ILD patients already using ambulatory oxygen, a topic guide will address a series of issues identified as important, including practical barriers to optimal oxygen usage, practical, social and psychological difficulties encountered, concerns about dependency, and views on the information required prior to ambulatory oxygen prescription. The interviews will also explore patients' (and carers') experience of participating in the trial and recruited at RBH in order to inform the definitive multi-centre trial. Field notes will be written after each interview to aid reflexive analytical processes (25). Individual feed-back on how the system and the service could be improved will assess how the needs of patients and their future involvement can be incorporated into the design of more patient centred devices.

17.3 Sample size and recruitment

17.3.1 Sample size calculation

As stated above, the primary outcome measure will be the change in total health status score of the K-BILD questionnaire that we have planned to collect every two weeks at each visit from V1 to V4 (14). Patients will be randomized to ambulatory oxygen or not, crossing over to the alternative arm after two weeks. The repeatability of the K-BILD questionnaire at

two weeks in patients with stable ILD is high, with an intra-class coefficient of 0.94 for the total health status score. This study is powered to detect a change of 8 units in the K-BILD total score, the minimally clinically important difference (SD: 20), recently estimated by using a range of distribution methods and anchoring K-BILD change against change in FVC and other established respiratory questionnaires (26).

We calculated that a sample size of 80 patients would allow 90% power at 5% significance to detect a difference of at least 8 points between the two crossover trial arms, allowing for a 15% drop out rate. This degree of power will also allow us to perform some exploratory subgroup analyses. For example, sub analysis of IPF patients only will be possible with adequate power. If we consider that IPF patients form 70% of the sample, this number of IPF patients (56) would still provide a power of 84% to detect a difference of 8 in the K-BILD health status score.

The software used for power calculations was "PS Power and Sample Size calculator version 3.0 by William Dupont and Walton Plummer 2009".

17.3.2 Planned recruitment rate

Recruitment of 80 patients with fibrotic ILD will occur over an 18 month period. Based on review of current practice, we are confident of recruiting 50 patients at RBH, and 15 each at KCH and Aintree over this period. IPF patients currently in the Profile study ("prospective observation of fibrosis in the lung clinical endpoints NCT01110694; > 100 currently enrolled) can enrol in this study.

17.4 Statistical analysis plan

17.4.1 Summary of baseline data and flow of patients

As the design of the study is a crossover trial, there will be no baseline comparison. Summary statistics will be presented to reflect the type of data, with categorical presented as %, and numerical data as mean ±SD or median with interquartile range, as appropriate. Some numeric variable will be presented both as continuous and as categorical based on known relevant cut-offs or thresholds.

A consort-statement diagram will be presented.

17.4.2 Primary endpoint analysis

The primary endpoint will be the change in K-BILD health status score. The mean change vs baseline in the K-BILD health status score on and off ambulatory oxygen will be compared by using either a paired t-test or Wilcoxon signed rank test, as appropriate to the distribution of the data. To allow assessment of the effect of baseline values on treatment and any other potential confounders, regression with robust variances or linear mixed models will be used. STATA software will be used for statistical analysis.

Predefined subgroup analysis will include an assessment of whether patients with elevated BNP and/or evidence of pulmonary hypertension on echo re more likely to benefit from ambulatory oxygen. Also patients with IPF which we expect to be the largest subgroup will

be analysed separately. IPF patients tend to experience the most severe desaturation on exertion and may therefore respond differently to supplemental oxygen than other fibrotic ILDs.

We expect missing data to be low. All data will be checked for validity and where data is questionable the appropriate investigator will query and resolve the inconsistency. For outlier values believed to be correct, a sensitivity analysis will be performed with and without such values. Subgroup analysis will follow the same analysis used for the primary outcome.

Our primary analysis will be based on intention to treat.

Qualitative methods: For the qualitative interview analysis, interviews will be transcribed verbatim. Interview transcripts will be analysed thematically using a framework approach (21). Through the five steps of (1) familiarisation, (2) identifying thematic framework, (3) indexing, (4) charting, and (5) mapping and interpretation, framework provides a systematic approach to analysis useful for applied qualitative research and enhances visibility of analysis for policy-makers and practitioners. It is the method of choice for this study as it enables results to be produced relatively quickly to inform policy or practice, as well being useful for studies where data collection is more structured, and where analysis needs to be viewed/assessed by others in the team (20). Further, it is particularly useful for linking qualitative to quantitative data which would facilitate a mixed methods analysis of the broader dataset thus providing comprehensiveness and greater knowledge yield (21;27) making best use of the data collected. Atlas/ti computer software (http://www.atlasti.com) will be used to manage and index the data prior to charting, mapping and interpretation.

17.4.3 Secondary endpoint analysis

For all continuous variables we will use a paired t-test or a Wilcoxon signed rank test, as appropriate. For categorical variables, a Wilcoxon signed rank test or paired ChiSquare will be used as appropriate. To allow assessment of the effect of baseline values on treatment and any other potential confounders, regression with robust variances or linear mixed models will be used.

17.5 Randomisation

Simple block method randomization will be performed to determine the order of treatment. As the study is un-blinded, varying block sizes will not be required.

17.6 Interim analysis

There will be no planned interim analysis as the study duration is short, and we do not expect any significant undesirable outcomes.

18 Committees in involved in the trial

Trial Steering Committee (TSC) - provides overall supervision of the trial and ensures that it is being conducted in accordance with the principles of GCP and the relevant regulations. The Trial Steering Committee should agree the trial protocol and any protocol amendments and provide advice to the Investigators on all aspects of the trial. A Trial Steering Committee may have members who are independent of the Investigators, in particular an independent chairperson. Decisions about continuation or termination of the trial or substantial amendments to the protocol are usually the responsibility of the Trial Steering Committee.

19 Direct access to source data

The Investigator(s)/institution(s) will permit trial-related monitoring, audits, REC review, and regulatory inspection(s), providing direct access to source data/documents. Trial participants are informed of this during the informed consent discussion. Participants will consent to provide access to their medical notes.

20 Ethics and regulatory requirements

The Sponsor will ensure that the trial protocol, Patient Information Sheet (PIS), Informed Consent Form (ICF), GP letter and submitted supporting documents have been approved by the MHRA and a main Research Ethics Committee (REC), prior to any patient recruitment taking place. The protocol and all agreed substantial protocol amendments, will be documented and submitted for ethical and regulatory approval prior to implementation.

Before site(s) can enrol patients into the trial, the Principal Investigator must apply for Site Specific Assessment from the Trust Research & Development (R&D) and be granted written NHS R&D approval. It is the responsibility of the Principal Investigator at each site to ensure that all subsequent amendments gain the necessary approval. This does not affect the individual clinician's responsibility to take immediate action if thought necessary to protect the health and interest of individual patients (see section 12 for details of reporting procedures/requirements).

Within 90 days after the end of the trial, the CI and Sponsor will ensure that the main REC and the MHRA are notified that the trial has finished. If the trial is terminated prematurely, those reports will be made within 15 days after the end of the trial.

The CI will supply a summary report of the clinical trial to the MHRA and main REC within one year after the end of the trial.

21 Monitoring plan for the trial

The trial will be monitored according to the monitoring plan agreed and written by the Sponsor, based on the internal risk assessment procedure. Where appropriate the CI will be asked to complete a copy of the Sponsor's self-monitoring template. It is the responsibility of the CI to ensure this is completed and submitted to the RO every on request (see Study Monitoring Plan). It is the responsibility of the RO to determine the monitoring risk assessment and explain the rationale.

For multi-centre studies sponsored by RB&HFT the PI at each site will also be required to complete this self-monitoring template and return the form at the same frequency, to the RO in parallel for review. It is the RO's responsibility to ensure that any findings identified in a PI's monitoring report are actioned in a timely manner and any violations of GCP or the protocol reported to the RO immediately.

Any urgent safety measures at either the CI or a PI site must be reported by that site Investigator within 3 days, as per UK Regulations.

The CI will be provided with a copy of the study monitoring report during the Trial Initiation monitoring visit.

22 Finance

This study is funded by NIHR Research for Patient Benefit.

23 Insurance and indemnity

NHS bodies are liable for clinical negligence and other negligent harm to individuals covered by their duty of care. NHS Institutions employing researchers are liable for negligent harm caused by the design of studies they initiate. The provision of such indemnity for negligent harm should be stated to the participant.

24 Publication policy

Data ownership rights will lie with the institution sponsoring the trial.

25 Statement of compliance

The trial will be conducted in compliance with the protocol, Sponsor's Standard Operating Procedures (SOPs), GCP and the applicable regulatory requirement(s).

The study conduct shall comply with all relevant laws of the EU if directly applicable or of direct effect and all relevant laws and statutes of the UK country in which the study site is located including but not limited to, the Human Rights Act 1998, the Data Protection Act 1998, the Medicines Act 1968, the Medicines for Human Use (Clinical Trial) Regulations 2004, and with all relevant guidance relating to medicines and clinical studies from time to time in force including, but not limited to, the ICH GCP**,** the World Medical Association Declaration of Helsinki entitled 'Ethical Principles for Medical Research Involving Human Subjects' (2008 Version), the NHS Research Governance Framework for Health and Social Care (Version 2, April 2005).

This study will be conducted in compliance with the protocol approved by the REC and according to GCP standards and UK Clinical Trials Regulation. No deviation from the protocol will be implemented without the prior review and approval of the Sponsor and REC except where it may be necessary to eliminate an immediate hazard to a research subject. In such case, the deviation will be reported to the Sponsor and REC as soon as possible.

26 References

1) Wells AU et al. Functional impairment in lone cryptogenic fibrosing alveolitis and fibrosing alveolitis associated with systemic sclerosis: a comparison. Am J Respir Crit Care Med. 1997 May;155(5):1657-64.

2) Risk C et al. Exercise alveolar-arterial oxygen pressure difference in interstitial lung disease. Chest. 1984; 85(1):69-74.

3) Weitzenblum E et al. Pulmonary hemodynamics in idiopathic pulmonary fibrosis and other interstitial pulmonary diseases. Respiration. 1983; 44(2):118-27.

4) Charlie S et al. Pulmonary hypertension in interstitial lung disease. Curr Opin Pulm Med 2005; 11:452—455.

5) Bye PT, Anderson SD, Woolcock AJ, Young IH, Alison JA. Bicycle endurance performance of patients with interstitial lung disease breathing air and oxygen. Am Rev Respir Dis. 1982 Dec;126(6):1005-12.

6) Visca D, Montgomery A, de Lauretis A, Sestini P, Soteriou H, Maher TM, Wells AU, Renzoni EA. Ambulatory oxygen in interstitial lung disease. *ERJ* 2011, in press (October 2011)

7) Frank RC, Hicks S, Duck AM, Spencer L, Leonard CT, Barnett E. Ambulatory oxygen in idiopathic pulmonary fibrosis: of what benefit? Eur Respir J. 2012 Jul;40(1):269-70.

8) 2002. ATS statement: guidelines for the six-minute walk test. *Am.J.Respir.Crit Care Med.* 166:111-117.

9) Eaton, T., P. Young, D. Milne, and A. U. Wells. 2005. Six-minute walk, maximal exercise tests: reproducibility in fibrotic interstitial pneumonia. *Am.J.Respir.Crit Care Med.* 171:1150-1157.

10) Lama VN, Flaherty KR, Toews GB, Colby TV, Travis WD, Long Q, Murray S, Kazerooni EA, Gross BH, Lynch JP 3rd, Martinez FJ. 2003 Prognostic value of desaturation during a 6-minute walk test in idiopathic interstitial pneumonia. Am J Respir Crit Care Med;168:1084-90.

11) Arnold E, Bruton A, Donovan-Hall M, Fenwick A, Dibb B, Walker E. Ambulatory oxygen: why do COPD patients not use their portable systems as prescribed? A qualitative study. BMC Pulm Med. 2011 Feb 11;11:9.

12) Raghu G, Collard HR, Egan JJ, Martinez FJ, Behr J, Brown KK, Colby TV, Cordier JF, Flaherty KR, Lasky JA, Lynch DA, Ryu JH, Swigris JJ, Wells AU, Ancochea J, Bouros D, Carvalho C, Costabel U, Ebina M, Hansell DM, Johkoh T, Kim DS, King TE Jr, Kondoh Y, Myers J, Müller NL, Nicholson AG, Richeldi L, Selman M, Dudden RF, Griss BS, Protzko SL, Schünemann HJ; on behalf of the ATS/ERS/JRS/ALAT Committee on Idiopathic Pulmonary Fibrosis. An Official ATS/ERS/JRS/ALAT Statement: Idiopathic Pulmonary Fibrosis: Evidence-based Guidelines for Diagnosis and Management. Am J Respir Crit Care Med. 2011 Mar 15;183(6):788-824.

13) Bradley B, Branley HM, Egan JJ, Greaves MS, Hansell DM, Harrison NK, Hirani N, Hubbard R, Lake F, Millar AB, Wallace WA, Wells AU, Whyte MK, Wilsher ML; British Thoracic Society Interstitial Lung Disease Guideline Group, British Thoracic Society Standards of Care Committee; Thoracic Society of Australia; New Zealand Thoracic Society; Irish Thoracic Society. Interstitial lung disease guideline: the British Thoracic Society in collaboration with the Thoracic Society of Australia and New Zealand and the Irish Thoracic Society. Thorax. 2008 Sep;63 Suppl 5:v1-58.

14) Patel AS, Siegert RJ, Brignall K, Gordon P, Steer S, Desai SR, Maher TM, Renzoni EA, Wells AU, Higginson IJ, Birring SS. The development and validation of the King's Brief Interstitial Lung Disease (K-BILD) health status questionnaire. Thorax. 2012 Sep;67(9):804-10.

15) Eakin EG, Resnikoff PM, Prewitt LM, Ries AL, Kaplan RM 1998. Validation of a new dyspnea measure: the UCSD shortness of breath questionnaire. Chest; 113:619-624.

16) Yorke J et al. Development and validity testing of an IPF-specific version of the St George's Respiratory Questionnaire. Thorax. 2010;65(10):921-6.

17) Nishiyama et al. Health-related quality of life in patients with idiopathic pulmonary fibrosis. What is the main contributing factor?. Respir Med. 2005; 99(4):408-14.

18) Zigmond, A. S. & Snaith, R. P. The hospital anxiety and depression scale. Acta Psychiatr. Scand. 67, 361-70 (1983).

19) Jaeschke R, Singer J, Guyatt GH. Measurement of health status. Ascertaining the minimal clinically important difference. Control Clin Trials. 1989 Dec;10(4):407-15.

20) Ritchie J, Spencer L. Qualitative data analysis for applied policy research. In: Bryman A, Burgess R G, eds. Analyzing qualitative data. London: Routledge; 1994. p.173-94.

21) O'Cathain A, Murphy E, Nicholl J. Why, and how, mixed methods research is undertaken in health services research in England: a mixed methods study. BMC Health Serv Res 2007;7:85. RfPB Competition 19 RfPB v1.0 Reference: PB-PG-0712-28073 Date submitted: 21/09/2012 Page 25 of 79

22) Hill K, Dolmage TE, Woon L, Goldstein R, Brooks D. Measurement properties of the SenseWear armband in adults with chronic obstructive pulmonary disease. Thorax. 2010 Jun;65(6):486-91.

23) Moore R et al. Comparison of pedometer and activity diary for measurement of physical activity in chronic obstructive pulmonary disease. J Cardiopulm Rehabil Prev. 2009 Jan-Feb;29(1):57-61.

24) Rabinovich RA, Louvaris Z, Raste Y, Langer D, Van Remoortel H, Giavedoni S, Burtin C, Regueiro EM, Vogiatzis I, Hopkinson NS, Polkey MI, Wilson FJ, Macnee W, Westerterp KR, Troosters T; PROactive Consortium. Validity of physical activity monitors during daily life in patients with COPD. Eur Respir J. 2013 Nov;42(5):1205-15.

25) Charmaz K. (2006) *Constructing Grounded Theory: A Practical Guide through Qualitative Analysis.* Sage Publications: London.

26) Patel AS, Siegert RJ, Keir GJ, Bajwah S, Barker RD, Maher TM, Renzoni EA, Wells AU, Higginson IJ, Birring SS. The minimal important difference of the King's Brief Interstitial Lung Disease Questionnaire (K-BILD) and forced vital capacity in interstitial lung disease. Respir Med. 2013 Sep;107(9):1438-43.

27) Barbour RS. The case for combining qualitative and quantitative approaches in health and services research. J Health Serv Res Policy 1999; 4:39-43.

27 Appendices

27.1 Appendix 1

Summary chart of study assessments

TIME	Screening Visit	2 WEEKS 'RUN IN' PERIOD	Treatment Visits		End of Treatment Visit
TIME	VISIT 1<br>WEEK - 2		Visit 2<br>Baseline & Randomization<br>Week 0	Visit 3<br>Cross over<br>Week 2	Visit 4<br>Week 4
Informed Consent	X				
Screening	X				
Eligibility Criteria	X				
Physical exam	X				
Blood pressure	X				
Pregnancy test	X				
Concomitant meds review	X		X	X	X
Randomization			X		
Study drug dispensing			X	X	
Cross over				X	
Adverse event checking			X	X	X
Routine bloods tests (Serum BNP)	X				
6 minute walking distance	X		x		
Spirometry	X				
Telephone calls	X		X	X	X
Oxygen Saturation and Heart Rate monitored continuously with Oxymeter WristOx2 3150			X		
6 MWT on air	X		X		
6 MWT on O_2	X		X		

**BORG Dyspnoea Scale before and after the 6MWT**	X		X		
**BORG Fatigue before and after the 6MWT**	X		X		
**Oxygen Saturation and Hear Rate before and after the 6MWT**	X		X		
**Time to recovery of oxygen saturation, heart rate, Borg dyspnea and fatigue**	X		X		
**Pulse oxiymeter** (at rest on RA*)	X		X	X	X
**Senswear Armband**[1]			X	X	
**Oxymeter**[2]			X	X	
**Global Assessment** of change in walking ability and breathlessness				X	X
**Diary of Daily Activities**			X	X	**COLLECT DATA FROM PATIENTS**
**K-BILD Questionnaire**	X		X	X	X
**SGRQ**			X	X	X
**SOBQ**			X	X	X
**HDAS Scale**			X	X	X
**Semi structured interviews for 20 patients**					X

*Grey shaded squares indicate tests performed as routine clinical care.

**Senswear Armband**[1]	Patient will be instructed to wear Senswear armband for 1 week during the 2 week treatment period (on air and on O_2 treatment arm)

**Oxymeter[2]**	Patients will be instructed to wear Oxymeter for 48 hrs during the 2 week treatment period (whilst on air or on O_2 treatment arm)

- BNP acceptable +/- 1 month before the screening visit.
- Spirometry and Echocardiogram acceptable within 6 months before the screening visit and up to 6 weeks after.
- 6MWT acceptable one week before or after the screening visit.
