# Supplementary figures and images for: Ambulatory oxygen in fibrotic lung disease (AmbOx): study protocol for a randomised controlled trial
Source: Trials. 2017 Apr 28;18:201. doi: 10.1186/s13063-017-1912-9 (PMC5410093; doi:10.1186/s13063-017-1912-9)

## Slide 1
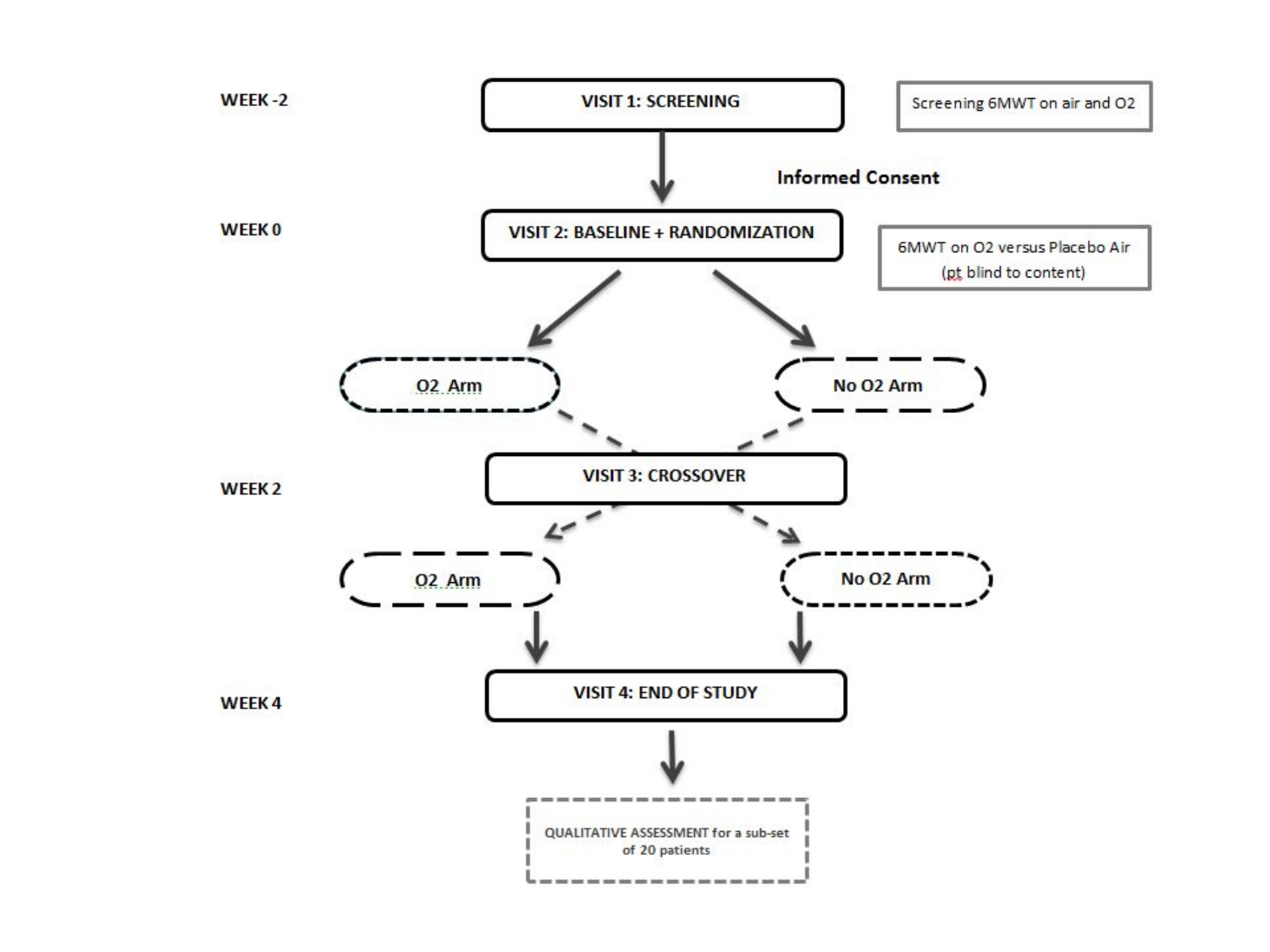

Supplement: Supplementary file 2 — Flow diagram. (PPTX 269 kb) [file 13063_2017_1912_MOESM2_ESM.pptx]
