# Supplementary material for: Ambulatory oxygen in fibrotic lung disease (AmbOx): study protocol for a randomised controlled trial
Source: Trials. 2017 Apr 28;18:201. doi: 10.1186/s13063-017-1912-9 (PMC5410093; doi:10.1186/s13063-017-1912-9)
Supplement: Supplementary file 3 — Patient Information Sheet: Version 3.0, 1 December 2014. (DOC 337 kb) [file 13063_2017_1912_MOESM3_ESM.doc]

| 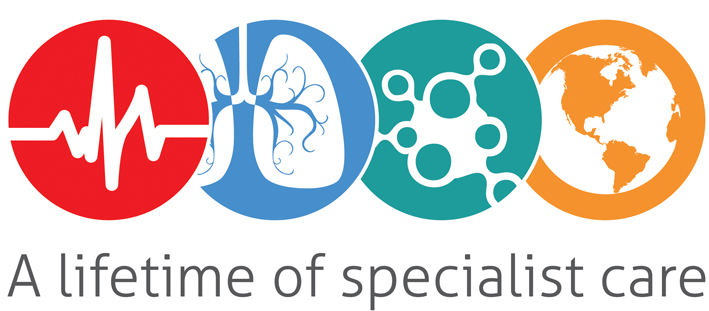 | 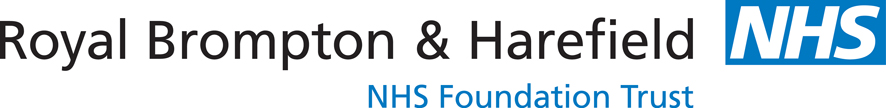 |
| --- | --- |

**Participant Information Sheet (PIS)**

**Study Title:** Randomized controlled, crossover trial to evaluate the Effects of Ambulatory Oxygen on health status in patients with Fibrotic Lung Disease (FLD)

**Short Study Title:** Ambulatory Oxygen in Fibrotic Lung Disease (FLD)

**Invitation to participate in the above study**

We invite you to participate in a research study for patients with Fibrotic Lung Disease (FLD: scarring of the lungs) who have a reduction in the level of oxygen in the blood on a six minute walk test (6MWT). This study will explore the effects of oxygen during periods of activity.

Before you decide whether or not you wish to participate in this study, it is important for you to understand why the research is being done and what it will involve. Please take the time to read the following information carefully and discuss it with others if you wish.

**What is the purpose of the study?**

To investigate if using oxygen during daily activities, such as walking, improves breathlessness and activity levels in patients with FLD. We will also investigate if the use of oxygen impacts upon other respiratory symptoms, and/or Quality of Life.

**Why have I been invited?**

You have been invited to participate in this study because you have fibrotic lung disease, defined by lung scarring and your oxygen saturation levels fall to or below 88% during a standard 6 MWT. We are planning to invite 80 patients with similar features to take part in the study.

**Do I have to take part?**

No. It is up to you to decide whether or not to take part. We will describe the study and go through this information sheet. If you agree to take part, you will be given this information sheet to keep and asked to sign a consent form. You are still free to withdraw at any time and without giving a reason.

Because your oxygen saturation drops to or below 88%, we will offer ambulatory oxygen to you as standard clinical practice. Although we think ambulatory oxygen is beneficial to patients with lung fibrosis, this has not been proven, and we do not know whether there are certain types of patients whom it benefits more than others.

**What will happen to me if I take part?**

If you agree to participate, you will be involved in the study for approximately six weeks. The initial screening visit will be conducted during your routine outpatient/inpatient assessment visit. You will be required to attend the hospital on three further occasions for research purposes only.

***Screening visit-visit 1***

You will undergo a number of examinations as part of your routine clinical care, which will include lung function tests, an echocardiogram and a six minute walk test (6MWT).

The 6MWT is a widely used test measuring exercise capacity. You will be asked to walk up and down a corridor for a total of six minutes. This walking test gives us important information on the degree of breathlessness and any drop in oxygenation levels you experience on walking. Normal values of oxygen saturation are ≥ 94%.

If your oxygen saturation drops to ≤ 88% during the 6 minute walking test, you will have a repeat 6MWT on supplemental oxygen (portable oxygen cylinder that can be carried around), to identify the oxygen flow needed to maintain optimal saturation

If you agree to take part in the study, we will ask you to complete a brief questionnaire about your breathing. If you are a woman of childbearing potential you will be tested for pregnancy.

It is necessary to ensure that your disease remains stable before you start with the oxygen treatment. The time between Visit 1 and Visit 2 is called ‘run in period’ .This period should last for two weeks and there should be no changes to your treatment or lifestyle in this period.

Following the two weeks ‘run in period’, you will be asked to return to the clinic to attend the second visit, Visit 2, called Baseline Visit.

***Baseline visit (or visit 2) and start of study treatment period***

We will check your oxygen saturation at rest on room air and your current medications. You will be asked to complete the same questionnaire about your breathing and three more questionnaires about breathing symptoms and quality of life. Filling in questionnaires should not take longer than 20 minutes in total.

You will complete a 6 minute walk test (6MWT) twice with a trained researcher with at least 20 minutes rest between tests. One 6MWT will be conducted with oxygen, and the other with an air-filled canister. The order of this will be random and we will not tell you which canister is which. The two tests should not take longer than 1 hour in total.

The study treatment period will last four weeks; two weeks will be spent using ambulatory oxygen and two on no oxygen (no portable devices). The allocation of this will be random. Only the order with which you will receive either oxygen or no oxygen will be randomized, the remaining part of your care will be identical during each of the two week periods. At the end of the baseline visit, your doctor will let you know if you will be using ambulatory oxygen for the following two weeks. If so at your third visit, your treatment will be changed to the no portable devices for a further two weeks and vice versa.

During the two weeks when you are allocated **oxygen**, you will be asked to use this oxygen during any activity that makes you breathless (such as walking, gardening, housework). For the two weeks when you are allocated **no oxygen,** you will be asked to carry out your normal day to day activities with no portable devices. All other aspects of your care will be consistent between the two periods. The study team will contact you by telephone during the four weeks of the study treatment period. Should you struggle with breathless in the two weeks on air and request oxygen cylinders, this would be arranged.

You will be also given an activity monitor, a small band to be placed around your arm that records the number of steps you take, and intensity of physical activity. You will be asked to wear this band during the second week of each two week period. We will also ask you to wear an oxymeter for 48 hrs in the week when you are wearing the armband. We will provide you with instructions. In addition to this, we will also ask you to complete a diary of daily activities.

Your participation in the study does not require modification of your regular medication, your lifestyle, or any dietary restrictions.

***Ambulatory oxygen treatment***

Ambulatory oxygen will be provided for you at home by the oxygen company operating within the location of your home. You doctor will order your supply of ambulatory oxygen as part of routine clinical care. Your oxygen prescription will specify the amount of oxygen required (hours per day) and flow rate. Ambulatory oxygen will be provided by the light weight oxygen gas cylinders. Overall there are four oxygen companies which provide supplemental oxygen in the UK. Light weight cylinders across the different oxygen companies provide oxygen flow rates from 0.5 to 15 L/min, lasting from 10 to 2 hours at a time depending on oxygen flow.. The oxygen will be administered through nasal cannula.

In order to arrange for oxygen to be delivered to your home, the research team will provide the supplier with details of your home address. The supplier will contact you to make arrangements for delivery, of the equipment and to train you to use the equipment. The supplier will also pass on your home address onto the fire brigade.

***Visit 3 - Cross Over visit***

On you arrival, we will check your oxygen saturation at rest on room air and your medications, as in visit 2. In addition, we will assess any adverse side effect of the oxygen therapy for patients’ allocated ambulatory oxygen during Visit 2. You will be asked to submit the armband and the oxymeter along with the completed diary of daily activities. You will be asked to complete the same set of questionnaires given to you during Visit 2.

You will also be asked to make an assessment of change in your walking ability and in your breathlessness on walking when compared to the previous 2 two weeks.

Following the assessment, your treatment will be switched to the alternative treatment for the next two weeks until visit 4. We will ask you to wear the armband and the oxymeter for the second of the following two weeks, and to continue with completing diary of daily activities as agreed at Visit 2. We will ask you to bring back measurements to Visit 4. Your doctor will arrange Visit 4 to take place at 2 weeks following the cross over visit.

***Visit 4 – End of treatment visit***

During the last study visit we will check your saturation at rest on room air, and your list of medications. We will ask you to submit the diary of daily activities, and return the armband and the oxymeter related to the final two weeks of study treatment. You will be asked to complete the same set of questionnaires given to you during visits 2 and 3. You will also be asked to make an assessment of change in your walking ability and in your breathlessness on walking when compared to the previous two weeks.

***Qualitative assessments***

A subgroup of individuals recruited at the Royal Brompton Hospital (RBH) will be asked to participate in the qualitative assessment aspect of the study. We would like to interview 20 patients and their careers. Interviews will be conducted by a member of the research team at the end of the study treatment period and are expected to last approximately half an hour, to gather information on patients’ and carers’ perspectives on the use of oxygen, its advantages and drawbacks. In particular, the domains which the interviews will focus on include the practical barriers to oxygen usage; practical, social and psychological difficulties encountered; concerns about dependency and views on the information required prior to ambulatory oxygen prescription. The interviews will also explore patients’ (and carers’) experience of participating in the trial. The interview will be tape recorded during the visit and the tape will be destroyed following the transcription of the information you provided.

***Blood Tests***

At screening visit we will undertake blood tests, which will take about 10ml (approximately 2 teaspoons) of blood by a trained person. The test for full blood count and serum BNP will be performed regardless of whether you choose to enter the study as part of routine clinical practice.

**Additional Visits**

If you are unwell you may require additional visits to hospital for your doctor to examine you and you may require tests, investigations and possibly treatment.

**Is the study a Randomised Trial?**

Yes. This is also a cross-over trial, so that each patient will have the different treatments (in this case oxygen or no oxygen) in turn, but the order with which this occurs will be assigned randomly (by chance).

**Expenses and payments**

You will be reimbursed for your time and reasonable travel expenses. Meals will be provided during the study visits.

**What do I have to do?**

We ask that you attend the hospital for all the clinic visits. You should tell your study doctor if you have any health problems, even if you think they are not caused by the study medication.

***Before agreeing to take part in this study you should check that any private medical insurance you have will not be affected by your participation.***

You must not take part in any other clinical study while you are participating in this study.

If you have agreed to take part in this study, you should:

- Follow the instructions provided by your study doctor and study staff.
- Tell your study doctor or study staff if you have received another treatment as part of a clinical trial within the previous 2 weeks.
- Tell your study doctor or study staff about any changes in your health or problems that you are having.
- Tell your study doctor or study staff about any medications you are taking even if they are obtained without a prescription.

**What are the alternatives for diagnosis or treatment?**

If the 6 minute walk test shows that your oxygen saturation falls to or less than 88% it means that during activities, the oxygen content in your blood is low. Oxygen supplementation on exertion can correct this gap, although there are no guidelines regarding the prescription of oxygen during daily activities.

**What are the possible disadvantages and risks of taking part?**

If the 6 minute walk test shows that your oxygen saturation falls to or less than 88%, we would be prescribing ambulatory oxygen regardless of participation to the study. However, as the need for ambulatory oxygen in lung fibrosis is not consistently evaluated across the UK, you will be asked to spend two of the four weeks not using ambulatory oxygen. This will not be different to what you have done up to now, and although it could lead to your being more breathless or being able to do less compared to when you will be using oxygen, we are unsure as to the extent of the benefit of ambulatory oxygen, and therefore a study is needed to compare symptoms with and without oxygen.

**What are the side-effects of any treatment received when taking part?**

The risks associated with the use of ambulatory oxygen are limited and highly manageable.

There is a theoretical risk of inappropriately high oxygen flows causing what in medical terms is called “hypercapnia”. There are two main gases in the human blood, oxygen and carbon dioxide. In some cases, patients have low oxygen (hypoxia) and high levels of carbon dioxide (hypercapnia). However, high carbon dioxide only occurs in some patients in the very severe stages of lung fibrosis, when low oxygen is also present at rest, and this group of patients will be excluded from the study.

In some cases the oxygen flow through nasal cannula could give mild local irritation to the nose, but this is unusual when oxygen is used only during activities. We do not expect you to experience any significant side effects from oxygen. However, if there are any problems or concerns outside of the study visits, you can contact us anytime by phone or email using contact details of your doctor provided in this Patient Information Sheet (PIS).

Ambulatory oxygen is associated with a potential fire hazard. However, this is well known and routinely managed in the context of the existing ambulatory oxygen service and all patients are educated with regards to the potential fire hazards of supplemental oxygen. In view of the potential fire hazard, current smokers will be excluded and your details will be given to the local fire brigades as outlined above.

**Radiation and the Ionising Radiation (Medical Exposure) Regulations – IRMER**

No additional x-rays, CT scans or exposure to radiation will occur as a result of taking part in this study.

**Pregnancy**

There are no know side and/or harmful effect of oxygen to suggest that oxygen adversely affects pregnancy and lactation. However, pregnant women will be excluded from the study. All female patients of childbearing potential must undergo a pregnancy test during the screening visit. In the case of a positive test, the patient will be excluded from enrolment.

**What are the possible benefits of taking part?**

This study aims to further the understanding of the ways in which ambulatory oxygen may be helpful to patients with lung fibrosis. It may also help you personally to formally assess whether ambulatory oxygen reduces your breathlessness and/or increases what you can do on a daily basis.

**What happens when the research study stops?**

After the study finishes, you will be able to continue with ambulatory oxygen and to attend the hospital for follow up visits according to your clinical care, as you have done until now.

Any doubts about its use can be discussed between with your treating doctor.

**What if there is a problem?**

If you have a concern about any aspect of this study and wish to complain formally, please contact Patient Advisory Liaison Service (PALS) if you have any concerns regarding the care you have received, or as an initial point of contact if you have a complaint. Please telephone Mrs Eve Cartwright on 0207 3528121 x2803 or email [e.cartwright@rbht.nhs.uk](mailto:e.cartwright@rbht.nhs.uk). You can also visit PALS by asking at any hospital reception.

**Will my taking part in the study be kept confidential?**

Any identifiable information that is collected about you in connection with this study will remain confidential and will be disclosed only with your permission, or except as required by law. If you join the study, your medical records and the data collected for the study may be reviewed by authorised persons from the Royal Brompton & Harefield NHS Foundation Trust who are the Sponsor of the study and are organising and managing the study.

They may also be reviewed by authorised persons from the UK Medicines Healthcare Regulatory Authority (MHRA) or other NHS organisations where it is relevant to your taking part in research to check that the study is being carried out correctly. All personnel will have a duty of confidentiality to you as a research participant. Any information about you which leaves the hospital will have your name, address and personal details removed so that you cannot be recognized from it and will be identifiable to only the study team.

**Will my General Practitioner / Family doctor (GP) be informed of my involvement?**

With your consent your GP will be informed that you are participating in the study and kept informed of your medical progress. We may exchange information regarding your general medical health with your GP.

**What if relevant new information becomes available?**

Sometimes we get new information about the treatment being studied. If this happens your research doctor will tell you and discuss whether you should continue in the study. If you decide not to carry on, your research doctor will make arrangements for your care to continue. If you decide to continue in the study he/she may ask you to sign an agreement outlining the discussion.

**What will happen if I don’t want to carry on with this study?**

Your participation in this study is voluntary and you may withdraw from the study at any time without prejudice to your future medical care. Should you decide to withdraw from the study for any reason, you are asked to contact the study team immediately. Should your participation in the study be terminated, regardless of the reason, you will not suffer any penalties or loss of benefits to which you are otherwise entitled.

If for any reason you decide to withdraw from the study earlier than planned the study Sponsor will wish to keep the data that has already been collected and the study team may ask for your permission to contact you, your GP or hospital medical team periodically to gather general information about your health after stopping the study treatment. Although you are under no obligation to give the reasons for stopping study treatment it may help the study team or benefit others if this information is made available.

If you are harmed as a direct result of taking part in this study, there are no special compensation arrangements. If you are harmed due to someone’s negligence, then you may have grounds for legal action. Regardless of this, if you have any cause to complain about any aspects of the way you have been approached or treated during the course of this study, the normal National Health Service (NHS) complaints mechanisms are available to you.

**What will happen to the results of the research study?**

If you give us your permission by signing the consent document, we plan to discuss/publish the results, by presenting them at conferences and sending them for consideration of publication to peer-reviewed journals. In any publication, information will be provided in such a way that you cannot be identified. We will also send a summary of the results to all the participants to this study.

**Who is organising and funding the research?**

The study is being organised and sponsored by the Royal Brompton and Harefield NHS Foundation Trust (RB&HFT). The study is funded by the National Institute of Health Research (NIHR) for Patient Benefit funding award.

**Who has reviewed the study?**

All research in the NHS is looked at by independent group of people, called a Research Ethics Committee (REC), to protect your interests. This study has been reviewed and given favorable opinion by NRES Committee London - Fulham.

**Further Information and Contact Details**

Should you have any further question about this research study, you may find it helpful to contact your doctor.

**IF YOUR DOCTOR IS NOT ACCESSIBLE FOR ANY REASON AN ALTERNATE SHOULD BE CONTACTED IMMEDIATELY:**

Dr Elisabetta Renzoni

Consultant Physician

Royal Brompton Hospital

Fulham Wing, Level M

SW3 6LR

0207 3528121 ext 8327

Dr Dina Visca

Clinical Research Fellow

Royal Brompton Hospital

Sydney Street

SW3 6LR

0207 3528121 ext 8327

Email address: [D.Visca@rbht.nhs.uk](mailto:D.Visca@rbht.nhs.uk)

**Thank you for taking the time to consider this study. If you do choose to participate, you will be given a copy of this information sheet to keep and also a copy of the consent form that you will be asked to sign.**
