# Supplementary material for: Ambulatory oxygen in fibrotic lung disease (AmbOx): study protocol for a randomised controlled trial
Source: Trials. 2017 Apr 28;18:201. doi: 10.1186/s13063-017-1912-9 (PMC5410093; doi:10.1186/s13063-017-1912-9)
Supplement: Supplementary file 4 — Informed Consent Form: Version 3.0, 1 December 2014. (DOC 32 kb) [file 13063_2017_1912_MOESM4_ESM.doc]

*(Insert on local Trust headed paper)*

**REC Reference Number:** Please insert REC Ref number

**Patient Identification Number for this trial: ­­­­________**

**INFORMED CONSENT FORM**

**Title:** Randomised, controlled crossover trial to evaluate the Effects of Ambulatory Oxygen on health status in patients with Fibrotic Lung Disease (FLD)

**Name of Researcher:** Please insert researcher name and address

**Please initial box**

1. I confirm that I have read and understand the information sheet dated 01^st^ December 2014 (Version 3.0) for the above study and have had the opportunity to consider the information, ask questions and have these answered satisfactorily.

2. I understand that my participation is voluntary and that I am free to withdraw at any time, without giving any reason, without my medical care or legal rights being affected.

3. I understand that relevant sections of any of my medical notes and data collected during the study may be looked at by responsible individuals from Royal Brompton and Harefield NHS Foundation Trust or from regulatory authorities where it is relevant to my taking part in research. I give permission for these individuals to have access to my records.

1. I agree to my GP being informed about this study and informed about any relevant results.
2. I agree to take part in the above study.

________________________ ________________ ____________________

Name of Patient Date Signature

_____________________ ________________ ____________________

Name of person taking consent Date Signature

***When completed 1 for patient; 1 for researcher; 1 (original) to be kept with hospital notes***
