# Supplementary material for: Ambulatory oxygen in fibrotic lung disease (AmbOx): study protocol for a randomised controlled trial
Source: Trials. 2017 Apr 28;18:201. doi: 10.1186/s13063-017-1912-9 (PMC5410093; doi:10.1186/s13063-017-1912-9)
Supplement: Supplementary file 5 — Exclusion criteria. (DOCX 15 kb) [file 13063_2017_1912_MOESM5_ESM.docx]

# Additional file 5

# Exclusion Criteria

- Age <18 or >99 years
- Patients expected to change treatment over the time course of the study
- Patients meeting criteria for long term oxygen therapy, i.e. hypoxic at rest (Oxygen Saturation at rest on room air <94%).
- Patients with significant communication or other locomotor difficulties, and/or severe co-morbidities.
- Patients with connective tissue disease-associated ILD or with sarcoidosis with musculoskeletal/joint involvement/symptoms
- Current smokers
- Pregnant women
- History of symptomatic ischaemic cardiac disease (exertion-induced chest pain)
- Anaemia, Hb < 9g/dl.
- Unable to provide informed written consent
